# Mitigating light pollution impacts on arthropods based on light‐emitting diode properties

**DOI:** 10.1111/cobi.70137

**Published:** 2025-08-24

**Authors:** Nicola van Koppenhagen, Martin M. Gossner, Jörg Haller, Janine Bolliger

**Affiliations:** ^1^ Swiss Federal Research Institute WSL Birmensdorf Switzerland; ^2^ EKZ Zurich Switzerland; ^3^ Department of Environmental Systems Science, Institute of Terrestrial Ecosystems ETH Zurich Zurich Switzerland; ^4^ Department of Geography University of Zürich Zurich Switzerland

**Keywords:** ALAN, arthropods, artificial light at night, ecological impact, ecosystem function, LED, light mitigation strategies, light pollution, sustainable outdoor lighting, artrópodos, contaminación lumínica, estrategias de mitigación lumínica, función ambiental, iluminación exterior sustentable, LAN, LED, luz artificial nocturna, 夜间人工光(ALAN), 可持续户外照明, 减缓光污染策略, 生态影响, 生态系统功能, 节肢动物, 发光二极管(LED), 光污染

## Abstract

Light pollution from artificial light at night (ALAN) is a significant environmental problem with far‐reaching consequences for ecological systems. Recent innovations in light‐emitting diode (LED) technology may offer sustainable outdoor lighting solutions, but scientific evidence is lacking. We investigated the effects of various LED lighting properties (color temperature, light intensity, and luminaire shape), individually and in combination, on flight‐active and ground‐dwelling arthropods. We therefore conducted a field experiment at 3 forest field sites in Switzerland with standardized LED streetlights. Over the course of 3 summers, we monitored flight‐active insects and ground‐dwelling arthropods with automated flight‐interception and pitfall traps. The absence of light reduced the number of arthropods caught by 91%. However, when lighting was necessary, dimming lights by 50% and using focused luminaires resulted in reductions of 22% and 42%, respectively. Light color influenced arthropod responses only when combined with dimming. Our results underscore the ecological benefits of darkness and the complex interactions among lighting properties. An optimized combination of these properties, particularly well‐focused and dimmed LED luminaires, represents a practical and effective measure to reduce the ecological impacts of ALAN and promote the conservation of nocturnal species.

## Abstract (es)

Mitigación del impacto de la contaminación lumínica sobre los artrópodos con base en las propiedades de los diodos emisores de luz

**Resumen:**

La contaminación lumínica provocada por la luz artificial nocturna (LAN) es un problema ambiental importante con consecuencias de gran alcance para los ecosistemas. Las recientes innovaciones en la tecnología de diodos emisores de luz (LED) pueden ofrecer soluciones sustentables para la iluminación exterior, pero faltan pruebas científicas. Investigamos los efectos de diversas propiedades de la iluminación LED (temperatura de color, intensidad luminosa y forma de la luminaria), tanto individualmente como en combinación, sobre los artrópodos voladores y terrestres. Para ello, realizamos un experimento de campo en tres bosques de Suiza con focos LED estandarizados. Durante tres veranos, monitoreamos los insectos voladores y los artrópodos terrestres con trampas automáticas de interceptación de vuelo y trampas de caída. La ausencia de luz redujo el número de artrópodos capturados en un 91%. Sin embargo, cuando era necesario iluminar, la reducción de la intensidad de la luz en un 50% y el uso de luminarias enfocadas dieron lugar a reducciones del 22% y del 42%, respectivamente. El color de la luz solo influyó en las respuestas de los artrópodos cuando se combinó con la atenuación. Nuestros resultados subrayan los beneficios ecológicos de la oscuridad y las complejas interacciones entre las propiedades de la iluminación. Una combinación óptima de estas propiedades, en particular luminarias LED bien enfocadas y atenuadas, representa una medida práctica y eficaz para reducir los impactos ecológicos de la LAN y promover la conservación de las especies nocturnas.

## 【摘要】

夜间人工光源(artificial light at night, ALAN)造成的光污染是一项严重的环境问题, 并对生态系统具有深远影响。近期关于发光二极管(light emitting diode, LED)技术的创新成果可能为户外照明提供可持续解决方案, 但尚且缺乏相关的科学证据。本研究分析了LED照明特性(色温、光强和灯具形状)对活跃飞行和地面栖息的节肢动物的影响, 包括这些特性的单独作用和组合作用。为此, 我们在瑞士三个森林实验站点用标准化LED路灯进行了野外实验。我们在三个夏季利用自动飞行拦截陷阱和坑式陷阱监测了活跃飞行的昆虫和地面栖息的节肢动物。在无光条件下, 捕获到的节肢动物数量减少了91%。然而, 当必须使用照明时, 将灯光调暗50%和使用聚焦灯具可分别导致捕获量减少22%和42%。色温仅在调暗亮度的情况下才会影响节肢动物的反应。我们的研究结果强调了黑暗的生态效益以及照明特性之间的复杂相互作用。这些特性的最优组合(特别是高度聚焦且调暗的LED灯具), 可作为一项实用且有效的措施, 来减少ALAN的生态影响并促进对夜行性物种的保护。**【翻译:胡怡思;审校:聂永刚】**

## INTRODUCTION

Arthropods are an integral part of the ecosystem and are essential providers of ecosystem services, including pollination, nutrient recycling, and pest control, and serve as a primary food source for various animals (Weisser & Siemann, 2013). However, there is increasing evidence of a global decline in arthropod numbers, species richness, and biomass (Hallmann et al., 2017; Wagner, 2019), a trend with significant ecological consequences. Although the decline in arthropods is largely attributed to land‐use change, habitat fragmentation, pesticide use, and climate change (Grubisic et al., 2018; Longcore & Rich, 2004; Seibold et al., 2019), there is also recent evidence that artificial light at night (ALAN) is an emerging and significant contributor to this decline (Linares Arroyo et al., 2024; Owens et al., 2020). ALAN is expanding globally at an alarming rate of 2–6% per year (Hölker et al., 2010; Kyba et al., 2023, 2017) and is considered a key driver of environmental change in the 21st century (Hölker et al., 2021).

ALAN can disrupt many natural processes, largely through attraction to artificial light sources—a mechanism referred to as positive phototaxis (Kim et al., 2019). Nocturnal arthropods, which are adapted to low‐light environments and exhibit, on average, 31% higher activity at night, are particularly susceptible to ALAN (Wong & Didham, 2024). The attraction to ALAN can disorient nocturnal insects, disrupt their natural behaviors, and increase their risk of exhaustion and predation. Over time, ALAN negatively affects their development, behavior, and fitness, ultimately leading to a deterioration in ecosystem functioning (Falcón et al., 2020; Kehoe et al., 2022). Although the impacts of ALAN on flight‐active arthropods are well documented (Owens & Lewis, 2018; Owens et al., 2020), comprehensive research on the overall effects on arthropod communities, for example, on flight‐active and ground‐dwelling arthropods, is mostly lacking (Manfrin et al., 2017). Because these 2 groups make important but distinct contributions to the functioning of our ecosystems, a nuanced understanding of light pollution impacts is crucial for developing effective mitigation strategies and prioritizing sustainable outdoor lighting solutions (Jägerbrand & Spoelstra, 2023).

Light design has undergone rapid technological advancements, which have led to light‐emitting diode (LED) lighting systems. They enable precise control of lighting properties, including spectral distribution, dimmability, luminaire shape, and color rendering options (De Almeida et al., 2014). There is evidence that these adjustable LED parameters may contribute to more sustainable lighting infrastructure. First, in terms of spectral composition, more sustainable lighting could be achieved by avoiding light with shorter wavelengths—such as those emitted by neutral‐white LEDs—because these have harmful impacts on arthropods (Brehm et al., 2021; Hao et al., 2023; Longcore, 2023). However, some studies have reported divergent impacts, suggesting that responses may vary depending on specific arthropod taxa or environmental conditions (Bolliger, Hennet, Wermelinger, Blum, et al., 2020; Kühne et al., 2021; Owens et al., 2022). Second, reducing the overall light intensity (dimming) significantly lowers the attraction to ALAN, making dimming outdoor lights a simple yet effective strategy to mitigate the ecological impacts of ALAN (Bolliger, Hennet, Wermelinger, Bösch, et al., 2020; Rowse et al., 2018). Third, the shape design of the luminaire is another critical but often overlooked factor. Light‐diffusing luminaire shapes attract more flight‐active insects (Bolliger et al., 2022), whereas well‐focused luminaires significantly reduce the number of attracted insects (Dietenberger et al., 2024). This evidence suggests that a targeted combination of adjustable LED and luminaire properties may significantly mitigate the environmental impacts of ALAN while maintaining safety and visual comfort for people. However, studies that combine adjustable LED light properties to investigate the impacts on flight‐active and ground‐dwelling arthropod communities are largely lacking.

We addressed this research gap by conducting a comprehensive experimental field study at 3 forest sites in Switzerland. We focused on the effects of LED streetlights on the abundance and composition of arthropod communities. We sought to assess the impacts of ALAN on flight‐active and ground‐dwelling arthropods (separately, combined, and by taxonomic group) by examining 3 key properties of LED lighting—color temperature, light intensity, and luminaire shape—and their interactions.

**FIGURE 1:**
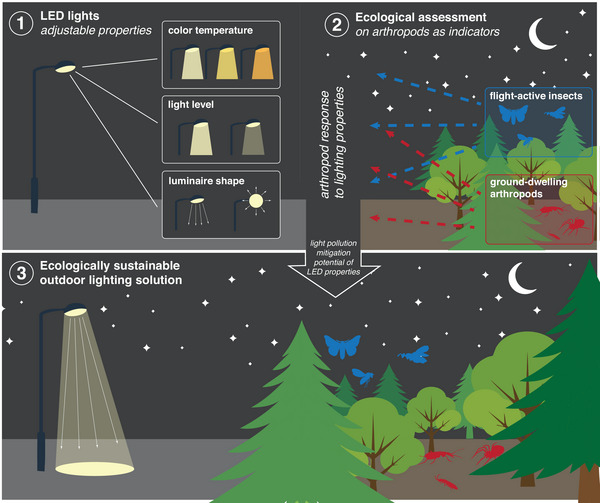
Conceptual framework of the assessment of the impacts of artificial light at night on the abundance and community composition of flight‐active insects and ground‐dwelling arthropods (based on different LED light properties: color temperature, light level, and luminaire shape).

By analyzing responses at the community level and across taxonomic groups, we aimed to provide critical insights into how modern LED lighting technologies can be designed to better protect nocturnal biodiversity and provide baseline information to guide the development of lighting solutions that contribute to conservation efforts by reducing ecological disruption and meet human needs (Figure 1).

## METHODS

### Study sites

Our study was conducted in 3 long‐term forest observation areas managed by the Swiss Federal Institute WSL (Swiss Federal Institute for Forest, Snow and Landscape Research) in Switzerland (https://lwf.wsl.ch/en/). The sites were in Birmensdorf (canton of Zurich, 47.363163, 8.454074), Lägern (canton of Aargau, 47.478771, 8.368333), and Alpthal (canton of Schwyz, 47.044189, 8.713174). All were supplied with electricity, equipped with meteorological stations, and not under the direct influence of ALAN prior to the study (Appendix S1).

The Birmensdorf site was in a forest area on the Swiss Plateau (1.13 ha, elevation 543 m asl, slope of ∼0%). The area features substrate from the Würm glaciation from the late Pleistocene. It is a managed temperate mixed deciduous forest in which European beech (*Fagus sylvatica*) is the dominant tree species. The mean annual temperature during the study period was 10.3°C, and the mean annual precipitation was 700 mm.

The Lägern site was in the community of Wettingen (1.34 ha, elevation 643 and 718 m asl, average slope 37%). The area is part of the Chain Jurassic and features substrates from the Jurassic. It is a managed temperate mixed deciduous forest in which European beech (*F. sylvatica*) is the dominant tree species. The mean annual temperature during the study was 10.4°C, and the mean annual precipitation was 1014 mm.

The Alpthal site was in the community of Alpthal (0.6 ha, elevation 1149 to 1170 m asl, mean slope 23%). The area is part of the Northern Pennsylvanian and Upper Cretaceous–Lower Eocene geological zones and has Wägital flysch substrates. The forest is temperate mixed conifer, and the dominant tree species is *Picea abies* (Norway spruce). The forest is managed for timber under a selective harvest system. The mean annual temperature during the study period was 7.0°C, and the mean annual precipitation was 1269 mm.

### Luminaire description and light treatments

We employed state‐of‐the‐art LED streetlight luminaires (Izylum 1, Schréder Swiss AG) to assess the effects of varying light properties on insect and arthropod communities. To ensure consistency across experimental treatments, the luminous flux of all luminaires was standardized to 1500 lumen, a calibration conducted at the Swiss Metrological Institute METAS in Bern (Appendix S29). The LED luminaires were configured to test 3 distinct light colors: neutral white (3700 K), warm white (2900 K), and amber (2200 K) (spectral distribution in Appendix S2). In addition, we evaluated the impact of 2 light intensity levels—full intensity (65 ± 6.5 lux at ground level) and 50% intensity (dimmed) (32.5 ± 3.25 lux at ground level). We also examined the effects of 2 luminaire shapes: a standard luminaire design with focused vertical light emission and a modified luminaire equipped with smooth‐white plexiglass tubes that diffused light more horizontally. The plexiglass tubes had a diameter of 150 mm, a length of 245 mm, and a light transmittance of 44% and were mounted directly under the light sources (Appendix S3).

At each of the 3 forest study sites in Switzerland, we installed 12 LED streetlights on aluminum poles at a height of 2.5 m. These poles were secured into the ground via ground screws (Krinner Schraubfundamente GmbH; KSF G 68×650‐3xM8). We established 2 dark control plots, which were set up identically to the streetlight plots but without light at night. This study design yielded a total of 36 streetlight plots and 6 control plots across all 3 sites (Appendix S1). To mitigate potential light spillover between treatments and ensure the integrity of our experimental design, streetlight plots were separated by a minimum of 30 m (Moran's *I* test was performed to confirm that there was no significant spatial autocorrelation [*I* = −0.016, *p* = 0.846] [Appendix S30]). The installation, ongoing monitoring, and maintenance of the LED systems were performed by technical experts from the Electricity Supplies of the Canton of Zurich (EKZ), which ensured that the light treatments were continuously and consistently applied throughout the study.

### Arthropod sampling

We conducted arthropod monitoring for flight‐active insects and ground‐dwelling arthropods over 3 consecutive summers (2021–2023). For flight‐active insects, the monitoring was carried out over a total of 12 weeks across 3 summers, 23 June–15 July in 2021, 8 June–30 June in 2022, and 7 June–29 June in 2023. Ground‐dwelling arthropods were monitored over 8 weeks across 2 summers, 23 June–15 July in 2021 and 8 June–30 June in 2022.

To collect flight‐active insects, we installed automated insect traps (Bolliger et al., 2020) on aluminum poles that were mounted directly on the 2.5‐m aluminum poles of the streetlights. The automated traps, designed to collect insects during specific periods, consisted of a turntable holding 7 cups that was rotated by a stepper motor powered by a rechargeable battery. The motor was programmed with a lookup table in the firmware that defines the duration of each time interval. The cups contained a 0.5% biocide solution (Rocima GT), which facilitated the sinking of insects and prevented the fluid from rotting in warm weather. The traps with the cups inside were covered with a lid with an attached aluminum funnel and an opening underneath. Each night, from sunset to sunrise, the turntable rotated to place a new cup under this funnel. The catching part of the automated insect trap was a commercially available flight‐interception trap (Polytrap [https://cahurel‐entomologie.com/shop/pieges/434‐piege‐d‐interception‐polytrap.html]). These traps consisted of 2 vertical, intersecting, transparent PET panels, which were fitted with a transparent roof and formed a barrier for flying insects in all directions. A funnel was attached under the panels, which led directly into the aluminum funnel of the automated trap below and captured insects that collided with the panels. The panels, roof, and funnel were made from flexible 1‐mm PET. The panels were 70 × 42 cm, and the funnel and roof had diameters of 45 cm. The Polytraps were securely attached directly to the streetlight heads, and each trap was positioned directly underneath the light source.

Ground‐dwelling arthropods were collected using standard pitfall traps (Lange et al., 2011). These funnel traps (diameter of 150 mm) were deployed at each streetlight approximately 1 m from the streetlight pole and were measured to receive 100% of the full light intensity (65 ± 6.5 lux measured at ground level). Each pitfall trap consisted of a plastic pipe (150 × 280 mm) buried in the ground into which a 750‐mL PVC bottle was inserted. A standard PVC funnel with a 150‐mm diameter was screwed onto the bottle and installed at the level of the ground surface. The bottles contained a 0.5% biocide solution (Rocima GT) to preserve the collected arthropods. These traps did not collect arthropods separately during the day and night.

The traps for flight‐active insects and ground‐dwelling arthropods were manually emptied every week during the sampling periods. The contents of each of the individual cups within the automated insect traps and each bottle of the pitfall traps were filtered through a paper tea filter (Cilia M). The tea filters containing the samples were then labeled and transferred into new sealable cups filled with 70% ethanol for preservation.

In the laboratory, the collected samples were identified and sorted into 41 arthropod groups under a stereomicroscope (Zeiss, Stemi 508). On the basis of this first initial sorting, the arthropods were further grouped at the order or suborder level (Formicidae being an exception at the family level and Neuropteroidea combining 3 orders [Neuroptera, Raphidioptera, and Megaloptera]). For the flight‐active insects, these groups were Nematocera, Brachycera, Lepidoptera, Coleoptera (Staphylinidae, Carabidae, other families), Hymenoptera (Apocrita, Symphyta), Hemiptera (Heteroptera, Sternorrhyncha, Auchenorrhyncha), Neuropteroidea (Neuroptera, Megaloptera, Raphidioptera), and Trichoptera. The ground‐dwelling arthropod groups included Collembola, Acarina, Formicidae, Arachnida (Araneae, Opiliones, Pseudoscorpiones), Coleoptera (predatory) (Carabidae, Staphylinidae), Coleoptera (other families), Myriapoda (Diplopoda, Isopoda, Chilopoda), and Hemiptera (Sternorrhyncha, Auchenorrhyncha, Heteroptera).

### Statistical analyses

All the statistical analyses were performed using R 4.2.2. We used linear mixed‐effect models (LMMs) (Bates et al., 2015) to assess the effects of color temperature, light intensity, and luminaire shape and their interaction on the abundance of various flight‐active insect and ground‐dwelling arthropod taxa and the sum of all arthropods. To do this, we followed a 2‐step approach. First, to assess arthropod attraction to light plots and control dark plots, each flight‐active insect taxa, the sum of all flight‐active taxa, each ground‐dwelling arthropod taxa, and the sum of all ground‐dwelling taxa were fitted independently as a function of a control term (control or no control). Light color (3 levels), light intensity (2 levels), and light shape (2 levels) were included as covariates. Two random effects were considered: week nested in year and plot nested in site.

Second, we excluded the control term (because the control is not fully factorial) to focus on the effects of the light treatments and the interactions between them. Therefore, each flight‐active insect taxa, the sum of all flight‐active taxa, each ground‐dwelling arthropod taxa, and the sum of all ground‐dwelling taxa were fitted independently as a function of light color (3 levels), light intensity (2 levels), light shape (2 levels), and all pairwise interactions between light properties (i.e., color × intensity, color × shape, intensity × shape) as fixed effects. The 2 random effects were week nested in year and plot nested in site.

The explanatory variables in the models were checked for overall model performance with the DHARMa package (Hartig et al., 2024) and for multicollinearity with the check_collinearity() function of the performance package (Lüdecke et al., 2021). Model performance was further assessed using *R* ^2^ (Bartoń, 2024) and the Akaike information criterion (AIC) for the full model. To ensure no significant spatial autocorrelation, Moran's *I* test was applied to the residuals from the LMM to assess spatial autocorrelation with a 40‐m distance threshold for defining neighbors in the spdep package (Bivand, 2022).

To assess and visualize the influence of environmental variables on community composition, we conducted a nonmetric multidimensional scaling (NMDS) analysis on the Bray‒Curtis dissimilarities with the vegan package (Oksanen et al., 2024). We performed permutational multivariate analysis of variance (PERMANOVA) with the adonis2 function to test the effects of light properties on community composition. The community matrix from the NMDS was used as the response variable, and light color temperature (3 levels), light intensity (2 levels), and light shape (2 levels) were used as explanatory variables. The analyses were performed separately for the flight‐active insects and ground‐dwelling arthropods.

## RESULTS

### Arthropod abundance

A total of 458,693 arthropods from 16 taxonomic groups were captured at 36 streetlights and 6 dark control plots, of which 64% were flight‐active insects and 36% were ground‐dwelling arthropods. The flight‐active insects consisted of Nematocera (70%), Coleoptera (8%), Lepidoptera (8%), Brachycera (5%), Hymenoptera (5%), Hemiptera (3%), Neuropteroidea (1%), and Trichoptera (1%). The ground‐dwelling arthropods consisted of Collembola (47%), Acarina (24%), Formicidae (11%), Arachnida (6%), Coleoptera (predatory) (4%), Coleoptera (other families) (4%), Myriapoda (3%), and Hemiptera (1%) (Appendix S4).

### Responses of arthropods to light exposure

**FIGURE 2:**
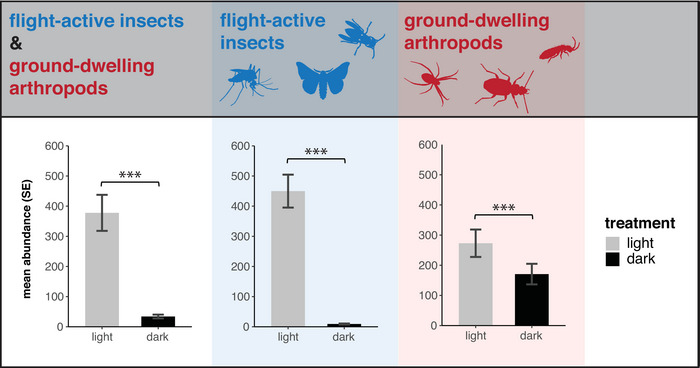
Abundance of flight‐active and ground‐dwelling arthropods in dark versus LED‐lighted areas (mean, estimated marginal mean; ***p < 0.001; **p < 0.01; *p < 0.05).

The mean abundance of arthropods collected was 91% lower in the dark controls than at streetlights with the lights turned on at night (Figure 2, left). Flight‐active insects were remarkably sensitive to light; abundance was 98% less under streetlights than it was in the dark (Figure 2, middle). Ground‐dwelling arthropod abundance was 37% less under streetlights than in the dark (Figure 2, right). The reduction in captured individuals in the dark controls was statistically significant for all flight‐active taxa and for 5 ground‐dwelling taxa (i.e., Collembola, Formicidae, Arachnida, Coleoptera [predatory], and Myriapoda) (Appendix S5). For detailed regression results, see Appendices S12–S17.

### Responses of arthropods to individual LED properties

**FIGURE 3:**
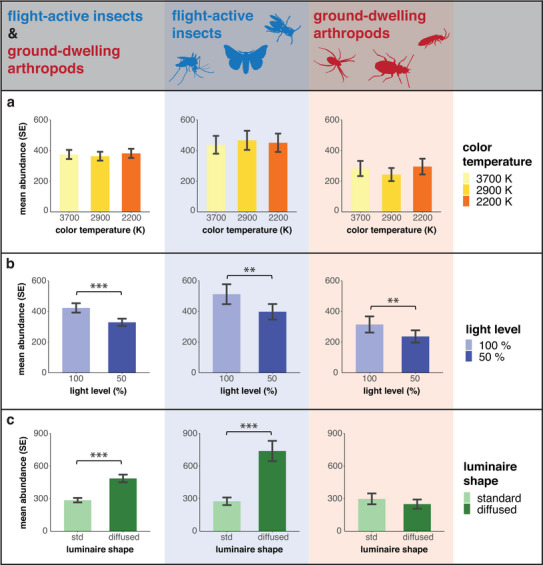
Impacts of LED properties (a) color temperature, (b) light level, and (c) luminaire shape on the abundance of flight‐active and ground‐dwelling arthropods (mean, estimated marginal mean; std, standard; ***p < 0.001; **p < 0.01; *p < 0.05).

Overall, flight‐active insects and ground‐dwelling arthropods were not sensitive to LED color temperature (Figure 3a). However, among individual taxa of flight‐active insects, 2 key pollinator taxa exhibited significant responses to light color temperature. Lepidoptera were 62% more abundant at 2900K sites than at 2200K sites, whereas Hymenoptera were 46% more abundant at 2200K sites than at 3700K sites (Appendix S6). Individual ground‐dwelling taxa did not respond significantly to the LED color temperature (Appendix S7).

Reducing the light intensity by 50% resulted in a significant 22% decrease in the mean number of collected arthropods (Figure 3b, left). Flight‐active insects were 22% and ground‐dwelling arthropods were 25% less abundant at luminaires set to 50% intensity than at luminaires set to full intensity (Figure 3b, middle & right). Among individual taxa of flight‐active insects, dimming to 50% intensity reduced the number of caught Nematocera (−25%), Brachycera (−19%), Coleoptera (−16%), and Hymenoptera (−25%) (Appendix S6). In ground‐dwelling arthropods, the numbers of caught Collembola (−21%) and Coleoptera (other families) (−35%) were significantly reduced with 50% light intensity (Appendix S7). The numbers of other taxa in both groups showed a general trend toward lower numbers under reduced light intensity, but these reductions were not statistically significant (Appendices S6 & S7).

Overall, the mean numbers of flight‐active insects and ground‐dwelling arthropods were significantly lower (−42%) when standard luminaires were used compared with luminaires that diffused light into the environment (Figure 3c, left). In particular, there were significantly fewer (−63%) flight‐active insects at well‐focused standard luminaires than at diffused luminaires (Figure 3c, middle). This was confirmed for all individual flight‐active taxa (Appendix S6). In contrast, luminaire shape did not significantly affect ground‐dwelling arthropods (Figure 3c, right; Appendix S7). Detailed regression results of the individual LED properties are in Appendices S18–S23.

### Responses of arthropods to interacting LED properties

**FIGURE 4:**
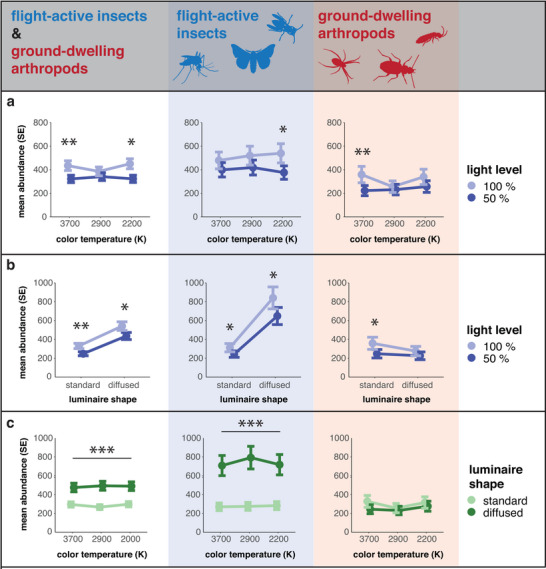
Impacts of LED (a) color temperature × light level, (b) luminaire shape × light level, and (c) color temperature × luminaire shape interactions (averages across treatments) on the abundance of flight‐active and ground‐dwelling arthropods (mean, estimated marginal mean; ***p < 0.001; **p < 0.01; *p < 0.05).

The mean number of arthropods caught was significantly lower at 2200 K (−28%) and 3700 K (−26%) when the light intensity was dimmed to 50% (Figure 4a, left). For flight‐active insects, the interaction effect of color temperature and light level was significant at 2200 K and 50% light intensity (−30%) (Figure 4a, middle) and for ground‐dwelling arthropods at 3700 K and 50% light intensity (−38%) (Figure 4a, right). No significant responses were observed for dimming at 2900 K (Figure 4a). We also detected taxon‐specific effects among flight‐active insects. The interaction between color temperature and light level resulted in significantly fewer Nematocera at 2200 K (−32%) and 3700 K (−26%) under 50% light intensity than at those temperatures under full intensity. The abundance of Hymenoptera and Coleoptera was significantly reduced only at 2200 K (−35% and −24%, respectively), whereas that of Brachycera was significantly reduced at 2900 K (−42%) (Appendix S8). In ground‐dwelling arthropods, the abundances of Collembola (−30%) and Arachnida (−55%) were significantly reduced at 3700 K when the light intensity was 50%. Acarina showed a reduction at 2200 K and 50% light intensity (−32%) and Coleoptera (other families) at 2900 K and 50% light intensity (−49%) (Appendix S9).

The mean overall number of arthropods for both luminaire shapes was lower when the light intensity was at 50% than when it was at full intensity (standard focus, −25%; diffused, −19%) (Figure 4b, left). For flight‐active insects, 50% intensity resulted in significantly lower arthropod abundances at both standard (−22%) and diffused luminaires (−23%) (Figure 4b, middle). In contrast, in ground‐dwelling arthropods, 50% intensity resulted in reduced numbers only at standard luminaires (−31%) (Figure 4b, right).

For individual taxa of flight‐active insects, a significant reduction was observed in Nematocera for both luminaire shapes (standard focus, −23%; diffused, −27%). In Coleoptera, 50% intensity significantly reduced the captured numbers only at standard luminaires (−24%), whereas for Hymenoptera, capture was significantly lower at diffused luminaires (−28%) (Appendix S8). For the ground‐dwelling taxa, significant reductions at dimmed luminaires were observed in Coleoptera (other families) for both luminaire shapes (standard focus, −33%; diffused, −37%) and in Formicidae for diffused luminaires (−70%) (Appendix S9).

Diffused light was significantly more attractive than standard‐focused luminaires to both arthropod groups, regardless of color temperature (2200 K, +64%; 2900 K, +85%; 3700 K, +61%) (Figure 4c, left). When analyzed separately, flight‐active insects appeared to be particularly susceptible to diffused luminaire shapes; numbers increased significantly (up to 189%) regardless of color temperature (Figure 4c, middle). In contrast, no significant effects of luminaire shape and color temperature were observed for ground‐dwelling arthropods (Figure 4c, right).

A significant increase in abundance was observed in all flight‐active taxa at diffused luminaires across all color temperatures, except Hymenoptera and Trichoptera (Appendix S8). Among the ground‐dwelling taxa, Collembola and Acarina numbers increased significantly at standard luminaires when the color temperature was 3700 K (+64%) and 2900 K (+44%), respectively (Appendix S9). For detailed regression results of the interacting LED properties, see Appendices S24–S26.

### Arthropod community composition as a function of LED properties

The community composition of flight‐active insects did not significantly differ among the 3 color temperatures (PERMANOVA, *F*_2, 380_ = 0.941, *R* ^2^ = 0.004, *p* = 0.444). However, light level (PERMANOVA, *F*_1, 380_ = 4.206, *R* ^2^ = 0.009, *p* = 0.006) and luminaire shape (PERMANOVA, *F*_1, 380_ = 80.425, *R* ^2^ = 0.172, *p* < 0.001) significantly influenced the order‐based insect community composition. However, the magnitude of the effect was subtle, as indicated by the low *R* ^2^ values and the overlap of the 95% ellipsoids (Appendix S10a). The slight variations in community composition in response to light level and luminaire shape were further illustrated by the mean proportional compositions of the insect groups (Appendix S10b). Detailed statistical results are in Appendix S27.

All 3 LED properties significantly affected the community composition of ground‐dwelling arthropods. The light level had the most substantial impact (PERMANOVA, *F*_1, 257_ = 6.531, *R* ^2^ = 0.024, *p* < 0.001), followed by the luminaire shape (PERMANOVA, *F*_1, 257_ = 3.384, *R* ^2^ = 0.012, *p* < 0.005) and color temperature (PERMANOVA, *F*_1, 257_ = 3.384, *R* ^2^ = 0.012, *p* < 0.011). However, the effect was marginal, as indicated by the low *R* ^2^ values and the considerable overlap of the 95% ellipsoids (Appendix S10a). The subtle changes in community composition between the light color temperatures, light levels, and luminaire shapes were visualized by the mean proportional compositions of the insect groups (Appendix S11b). Detailed statistical results are in Appendix S28.

## DISCUSSION

Our systematic and comprehensive study on the impacts of state‐of‐the‐art LED luminaires on arthropod communities provided new insights into ways to reduce the negative impact of light pollution, which could result in more sustainable outdoor lighting. Our results confirmed that the complete avoidance of ALAN is the most effective mitigation strategy, resulting in 91% fewer captured individuals. However, because turning off light is often not possible, our analyses of individual and combined LED properties provided critical insights into sustainable lighting strategies: dimming lights to 50% combined with standard, well‐focused luminaires reduced the number of flight‐active and ground‐dwelling arthropods caught at lights. Furthermore, the significant interactions between color temperature and luminaire shape with dimming highlighted the importance of considering the interaction between different LED light properties to mitigate negative impacts of ALAN and support the conservation of nocturnal biodiversity.

The effect of no lighting was particularly strong for flight‐active insects. There were 98% fewer individuals caught where there was no light than where there was light. This strong response, driven by positive phototaxis (Fabian et al., 2024), is consistent with previous research on the vulnerability of nocturnal insects to ALAN (Falcón et al., 2020; Firebaugh & Haynes, 2019; Gaston et al., 2015). This strong contrast between light and dark has a direct impact on insect populations, and because many nocturnal insects are key pollinators and prey for other animals, their strong attraction to ALAN could have cascading effects throughout the food web (Manfrin et al., 2018; Sánchez‐Bayo & Wyckhuys, 2019; Sullivan et al., 2019) and the ecosystem processes they support (Buxton et al., 2022; Knop et al., 2017; Macgregor & Scott‐Brown, 2020). Although ground‐dwelling arthropods were less sensitive to light exposure, significantly fewer were caught when there was no light (−37%), which is also in line with a previous study published on this group (Van Koppenhagen et al., 2024). The lower impact on ground‐dwelling taxa might reflect differences in exposure levels because many species are shielded by vegetation or terrain. However, the significant reduction observed in several taxa, such as detritivorous Collembola, omnivorous Formicidae, and predatory Coleoptera, suggests that ALAN can disrupt ecological processes below the shrub and herb layer, including decomposition and predation (Bennie et al., 2018; Brown et al., 2023; Hines & Gessner, 2012). Given that it is often not possible to turn off light completely due to human needs—such as the feeling of safety and visibility (Kaplan & Chalfin, 2022; Maier & DePrince, 2020)—these differentiated results regarding different facets of biodiversity in response to different LED properties are critical for identifying mitigation strategies to minimize ecological disruption while meeting human needs.

Our results revealed complex arthropod responses to specific LED properties. In contrast to many previously published articles that reported stronger attraction to shorter wavelengths (Brehm et al., 2021; Donners et al., 2018; Somers‐Yeates et al., 2013; Yang et al., 2024), overall arthropod abundance was not highly sensitive to changes in color temperature. There is limited evidence suggesting significant differences in arthropod attraction between neutral‐white and warm‐white LEDs, likely caused by marginal differences in the amount of light emitted in the blue spectrum (Bolliger et al., 2022; Hao et al., 2023; Owens et al., 2024; Van Koppenhagen et al., 2024). However, the response of individual flight‐active taxa—such as Lepidoptera and Hymenoptera—to certain color temperatures suggests that specific wavelengths could selectively affect key functional groups. Lepidoptera showed increased activity at cooler light temperatures (2900 K), whereas Hymenoptera were more active at warmer light temperatures (2200 K), highlighting the complexity of light spectrum interactions across taxa.

Reducing the light intensity by 50% effectively reduced the overall arthropod abundance by 22%, a finding that is consistent with previous studies on flight‐active insects (Bolliger, Hennet, Wermelinger, Bösch, et al., 2020; Owens & Lewis, 2018) and ground‐dwelling arthropods (Davies et al., 2017). These reduced abundances were consistent across the flight‐active and ground‐dwelling groups (−22% and −25%), with particularly strong effects on individual taxa such as Nematocera and Collembola. This suggests that dimming streetlights by 50% could serve as a highly effective mitigation strategy for minimizing the impact of ALAN on arthropods. However, because there is often a trade‐off between ecosystem‐friendly practices and human needs, further research on light intensities is essential. Light levels that are minimally acceptable to humans may still exceed thresholds that are tolerable for many nocturnal arthropods.

The shape of luminaires was particularly important for flight‐active insects: luminaires diffusing the light strongly into the environment increased the attraction of flight‐active insects by 63%, consistent with findings from previous studies that identified the dispersion of light as a critical factor in the attraction of flight‐active insects (Bolliger et al., 2022). In contrast to aerial insects, ground‐dwelling arthropods were unaffected by luminaire shape. Because they are active on the ground, they may not be directly affected by the light distribution of the light source at 2.5 m height, and the light reaching the ground by standard and diffusing luminaires may not be perceived differently by ground‐dwelling individuals. These findings suggest that replacing light‐diffusing luminaire shapes with targeted luminaires designed to reduce light scatter could be a highly effective strategy for minimizing the impact of light pollution on arthropods. This approach aligns with a recent study demonstrating that insect attraction decreases significantly when highly focused luminaires are used (Dietenberger et al., 2024).

The interactions between LED properties proved important for flight‐active and ground‐dwelling arthropods and revealed more differentiated effects than individual factors alone. Although light color temperature alone did not provoke a response, the combined effects of color temperature and dimming significantly reduced flight‐active insects under dimmed, warmer lights (2200 K). In contrast, ground‐dwelling arthropods experienced a reduction in cooler color temperatures (3700 K). No significant dimming effect for any arthropod group was observed for the intermediate color temperature of 2900 K. Additionally, we found no general impact of luminaire shape on ground‐dwelling arthropods, but dimming standard‐shaped luminaires resulted in a significant reduction in the number of captured animals. This finding complements the findings that flight‐active insects experience a strong positive phototactic response to diffused luminaires, and future lighting designs should therefore prioritize minimizing light diffusion or eliminating diffusing shapes to reduce arthropod and ecosystem disruption. These interactive effects of light properties emphasize the importance of considering multiple lighting parameters when designing sustainable outdoor lighting solutions. Standardized recommendations that account for these interactions could significantly reduce the negative impacts of ALAN.

Our results also highlighted that LED properties not only affect arthropod abundance but also alter arthropod community composition, resulting in subtle but significant shifts in flight‐active and ground‐dwelling communities. These shifts suggest that certain groups of arthropods are more sensitive to certain lighting conditions than others. For example, dimming and luminaire shape significantly altered the community composition of flight‐active and ground‐dwelling taxa. This underscores the importance of luminaire shape and dimming as 2 key LED properties that influence arthropod biodiversity. These subtle yet significant shifts in community composition carry the potential for long‐term ecological consequences, as shown in a previous study in which long‐term exposure to ALAN resulted in a decrease of macromoth abundance of over 14% (Van Grunsven et al., 2020). Given the integral role of arthropod communities in ecosystem functioning, even minor shifts in community composition could disrupt critical ecosystem services, such as pollination (Giavi et al., 2021; Knop et al., 2017; Wilson et al., 2021), pest control (Grubisic et al., 2018; Sanders et al., 2015), and nutrient flow (Manfrin et al., 2018).

Our results provide new insights into how ALAN affects flight‐active and ground‐dwelling arthropods through variations in LED properties. By demonstrating how color temperature, dimming, and luminaire shape—individually and in combination—affect arthropod communities, we provide new evidence that will form the basis for improved strategies to modify outdoor lighting practices and mitigate ecological disruption. The fact that dimming and more targeted use of luminaires can significantly reduce the attraction of nocturnal arthropods is a promising step toward the development of environmentally friendly lighting strategies. As lighting technologies continue to evolve, integrating such knowledge into lighting policy and infrastructure design is essential to safeguard nocturnal biodiversity and contribute to broader conservation goals.

## AUTHOR CONTRIBUTIONS

**Nicola van Koppenhagen**: Conceptualization (equal to Janine Bolliger); data curation; formal analyses (lead); investigation; methodology (supporting); project administration (equal to Janine Bolliger); visualization; writing—original draft. **Martin M. Gossner**: Conceptualization (supporting); formal analyses (supporting); methodology (equal to Janine Bolliger); resources (equal to Janine Bolliger); validation (equal to Janine Bolliger); writing—review and editing (equal to Janine Bolliger). **Jörg Haller**: Funding acquisition (supporting); resources (lead); validation; writing—review and editing (supporting). **Janine Bolliger**: Conceptualization (equal to Nicola van Koppenhagen); funding acquisition (lead); methodology (equal to Martin M. Gossner); project administration (equal to Nicola van Koppenhagen); resources (equal to Martin M. Gossner); supervision (lead); validation (equal to Martin M. Gossner); writing—review and editing (equal to Martin M. Gossner).

## Supporting information



Supporting Information
