# Supplementary material for: Mitigating light pollution impacts on arthropods based on light‐emitting diode properties
Source: Conserv Biol. 2025 Aug 24;40(1):e70137. doi: 10.1111/cobi.70137 (PMC12856788; doi:10.1111/cobi.70137)
Supplement: Supplementary file 1 — Supporting Information [file COBI-40-e70137-s001.pdf]

Supplementary materials

Supplementary Figures

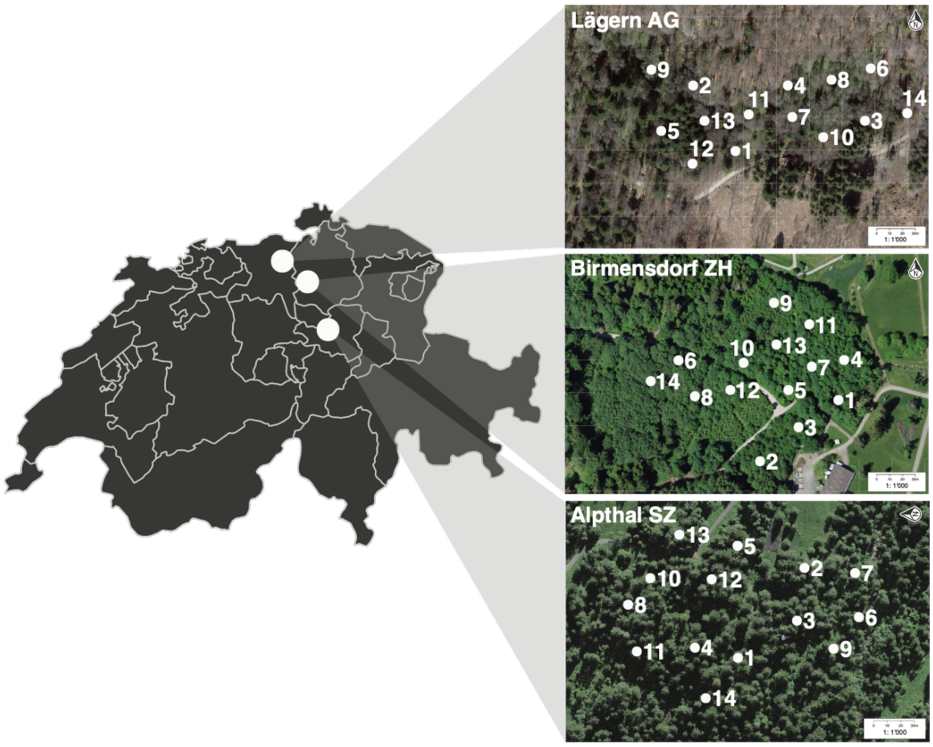

Light treatments

**1** 3700K / 100% / standard	**8** 2900K / 50% / diffused
**2** 3700K / 50% / standard	**9** 2200K / 100% / standard
**3** 3700K / 100% / diffused	**10** 2200K / 50% / standard
**4** 3700K / 50% / diffused	**11** 2200K / 100% / diffused
**5** 2900K / 100% / standard	**12** 2200K / 50% / diffused
**6** 2900K / 50% / standard	**13** no light
**7** 2900K / 100% / diffused	**14** no light

Figure S1. Three study sites and allocation of streetlight treatments.

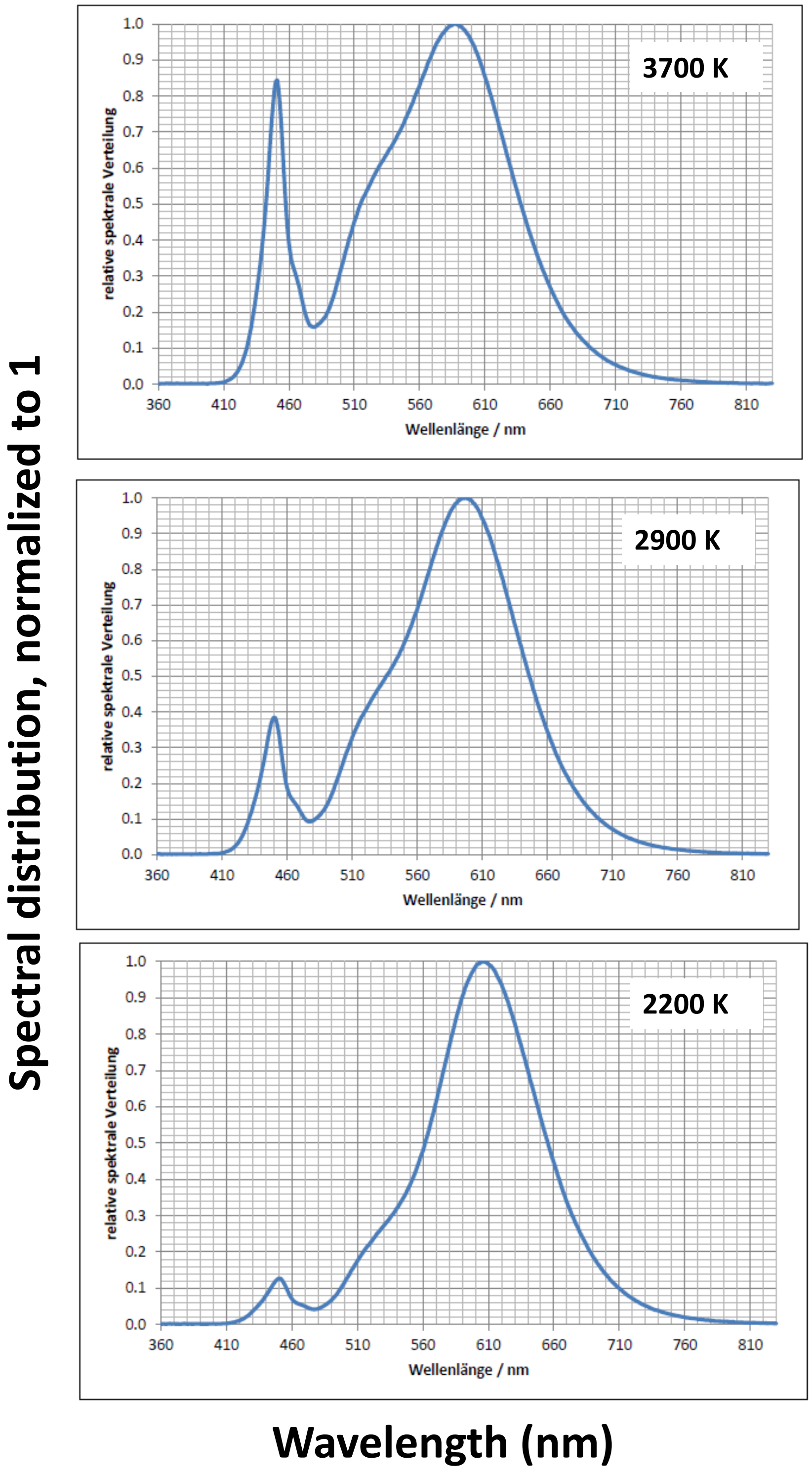

Figure S2. Spectral distribution covered by the three LED colors: 3700 K (top), 2900 K (center), and 2200 K (bottom).

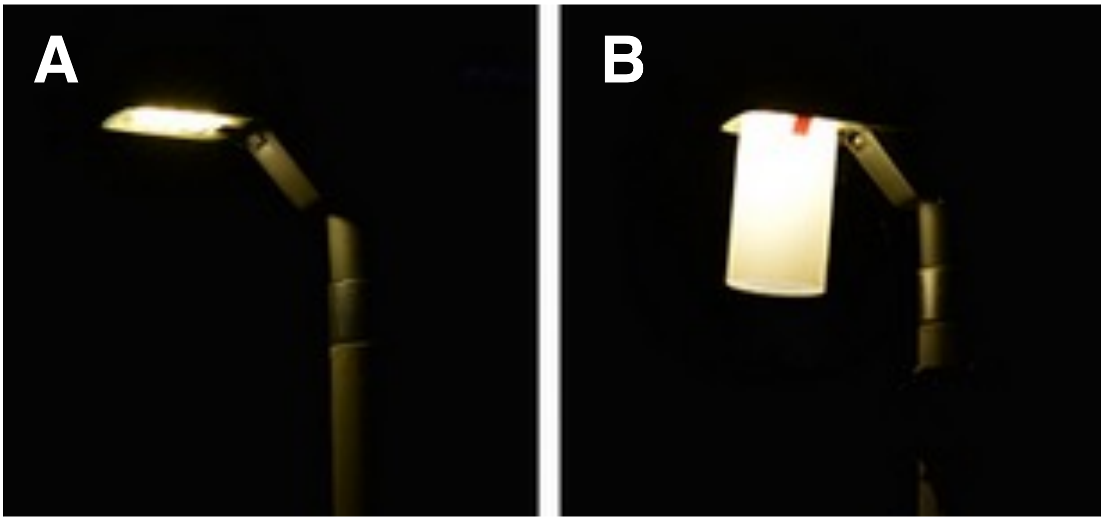

Figure S3. Luminaire shapes: A) Standard luminaire and B) luminaire with Plexiglas diffusor.

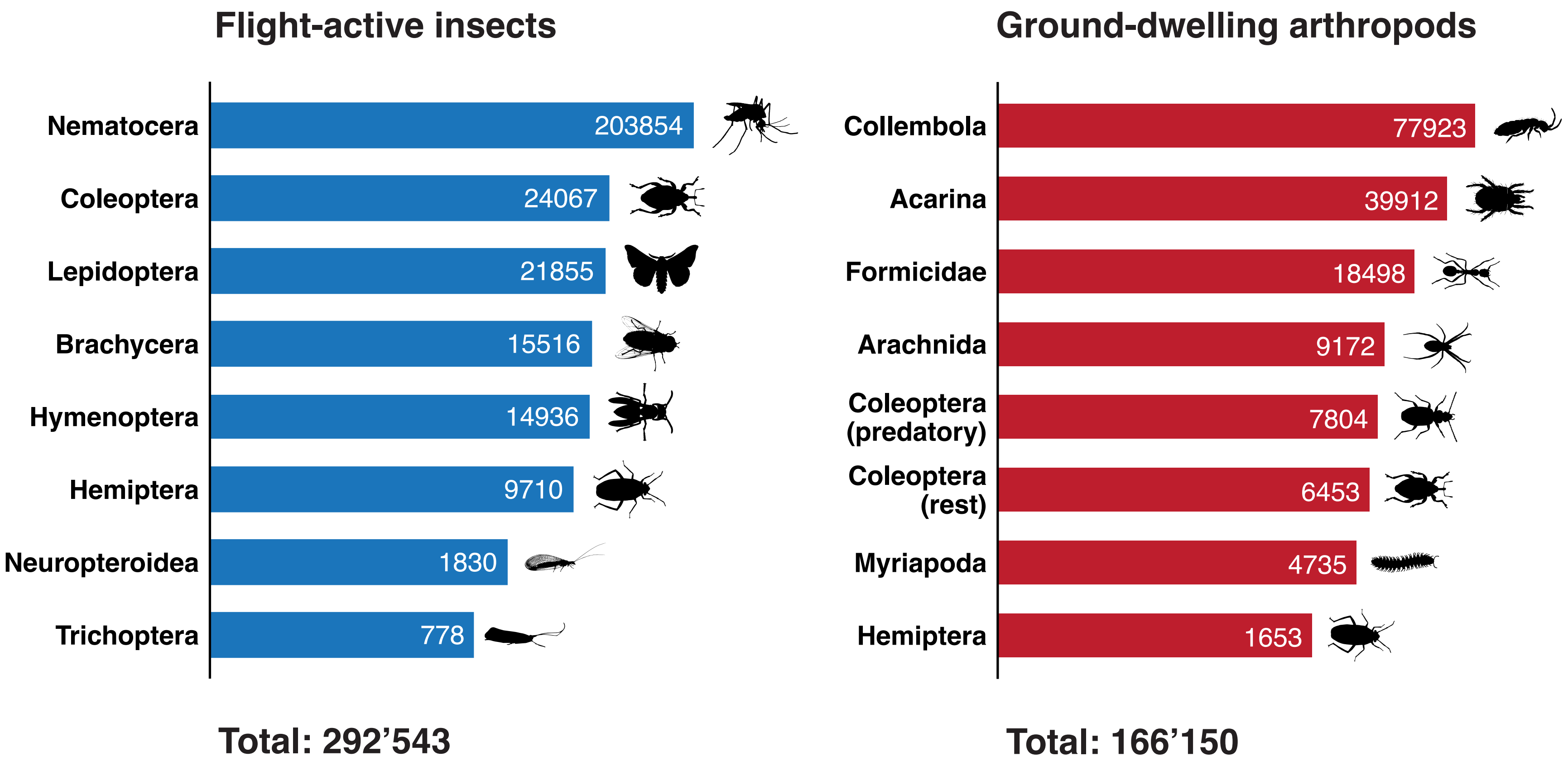

Figure S4. The total number of captured individuals of each arthropod taxonomic group separeted into two groups (flight-active insects, ground-dwelling arthropods) on a logarithmic scale (total number of caught individuals = white numbers inside the bar).

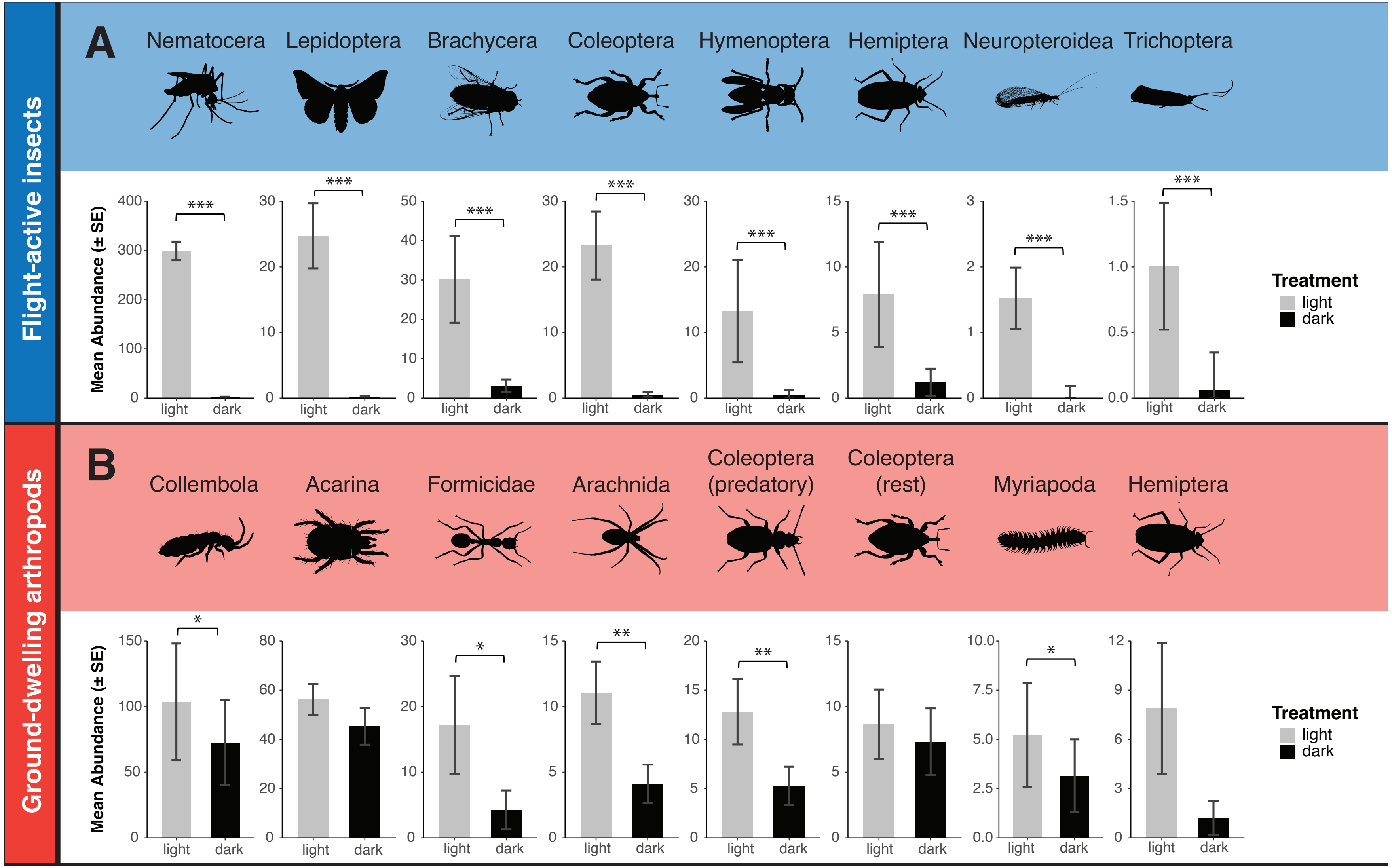

Figure S5. Estimated marginal means of the abundance of flight-active insect taxa and ground-dwelling arthropod taxa for two levels of light treatments (light, no light (dark)). The error bars show the standard error (SE). Significant results indicated by an asterisk (*) (*** $p<0.001$; ** $p<0.01$; * $p<0.05$).

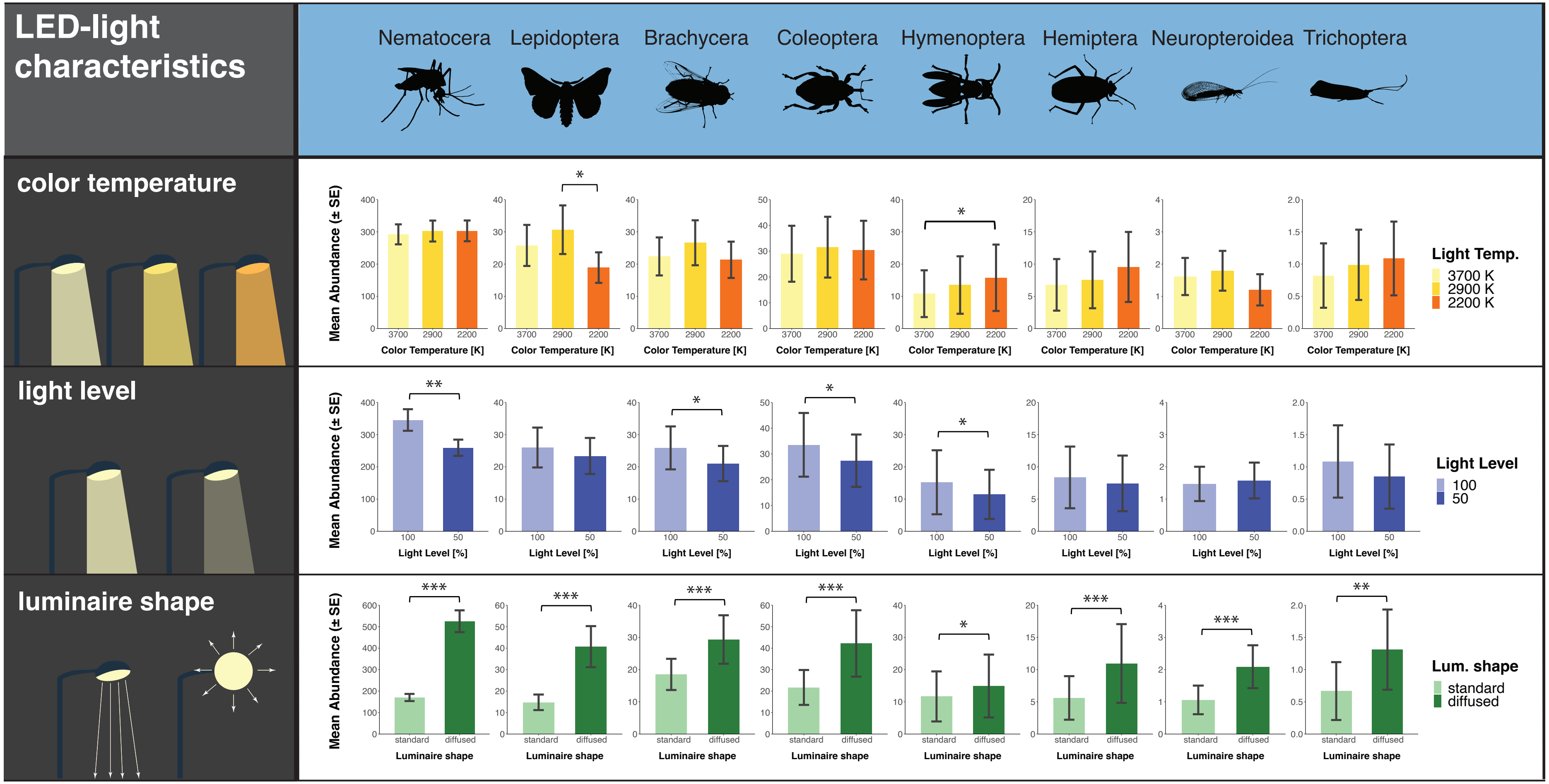

Figure S6. Estimated marginal means of the abundance of flight-active insect taxa for three LED color temperatures (A), two light levels (B), and two luminaire shapes (C). The error bars show the standard error (SE). The error bars show the standard error (SE). Significant results indicated by an asterisk (*) (*** p<0.001; ** p<0.01; * p<0.05).

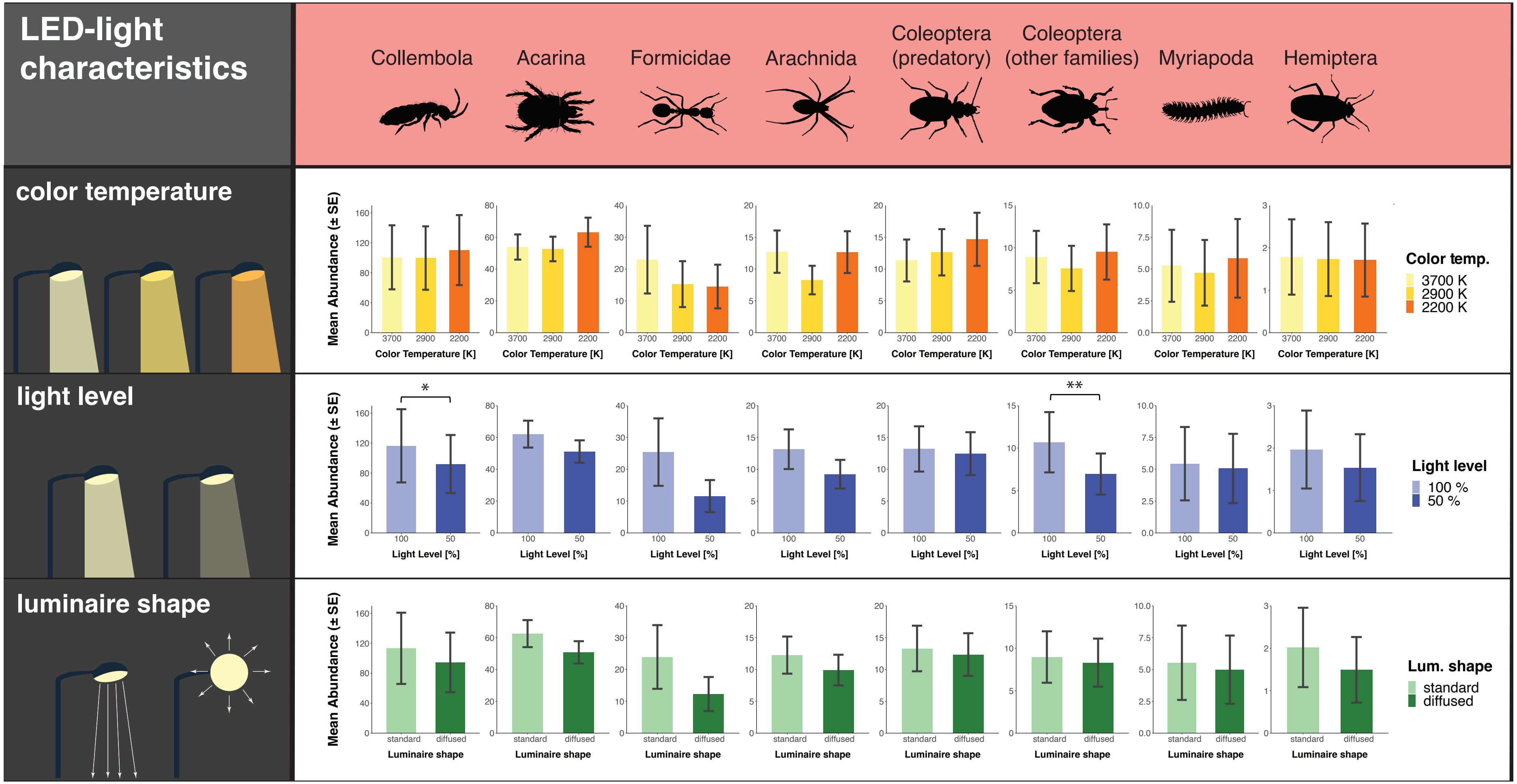

Figure S7. Estimated marginal means of the abundance of ground-dwelling arthropod taxa for three LED color temperatures (A), two light levels (B), and two luminaire shapes (C). The error bars show the standard error (SE). Significant results indicated by an asterisk (*) (*** p<0.001; ** p<0.01; * p<0.05).

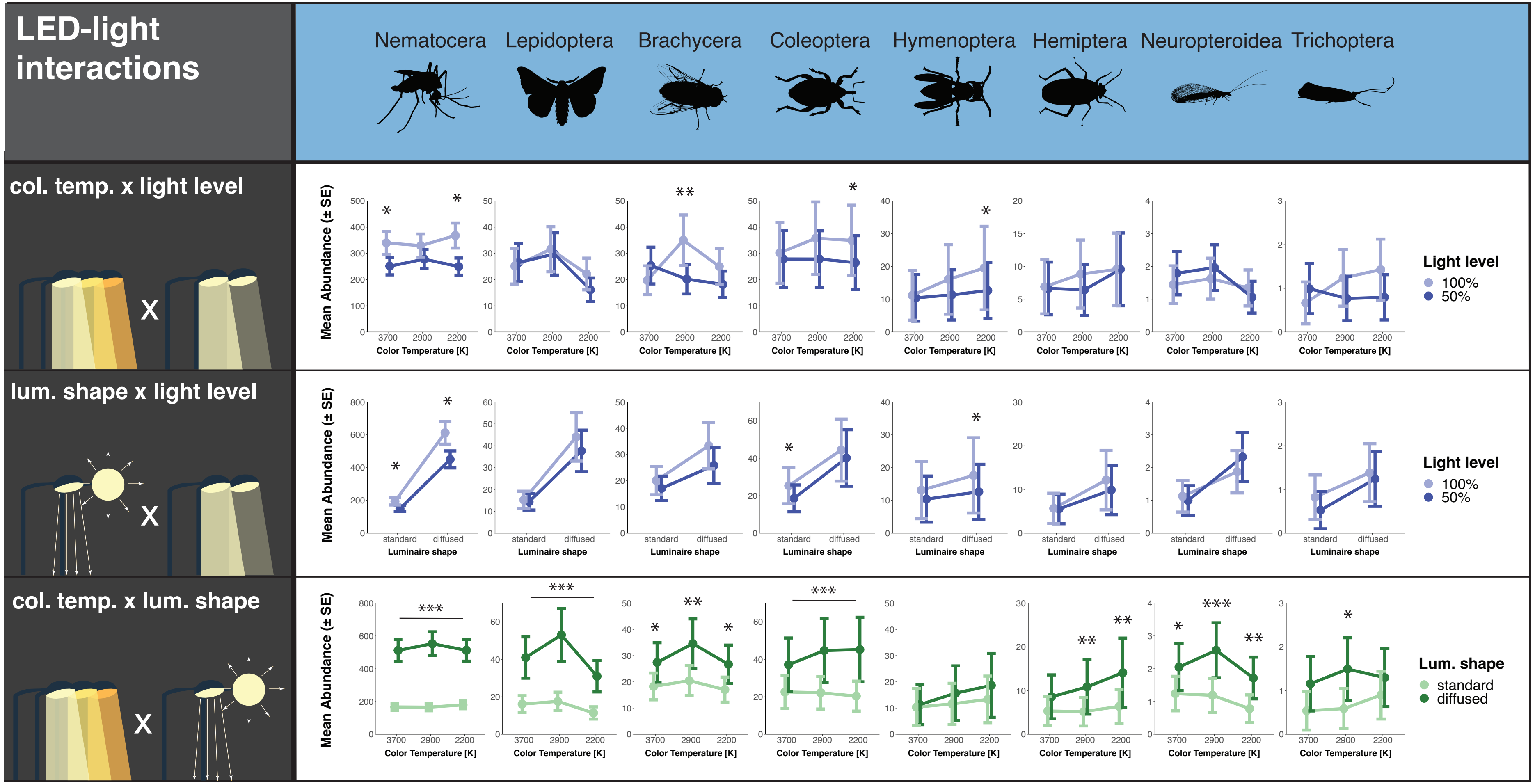

Figure S8. Estimated marginal means for the interactions of three luminaire characteristics (color temperature, light level, luminaire shape) on the abundance of flight-active insect taxa .The interactions between the light levels and temperatures averaged across both luminaire shapes (A); the interactions between the light levels and luminaire shapes averaged across the three color temperatures (B) and the interactions between the luminaire shapes and the color temperatures averaged across the two light levels (C). The error bars show the standard error (SE). Significant interactions (p<0.05) are indicated by an asterisk (*).

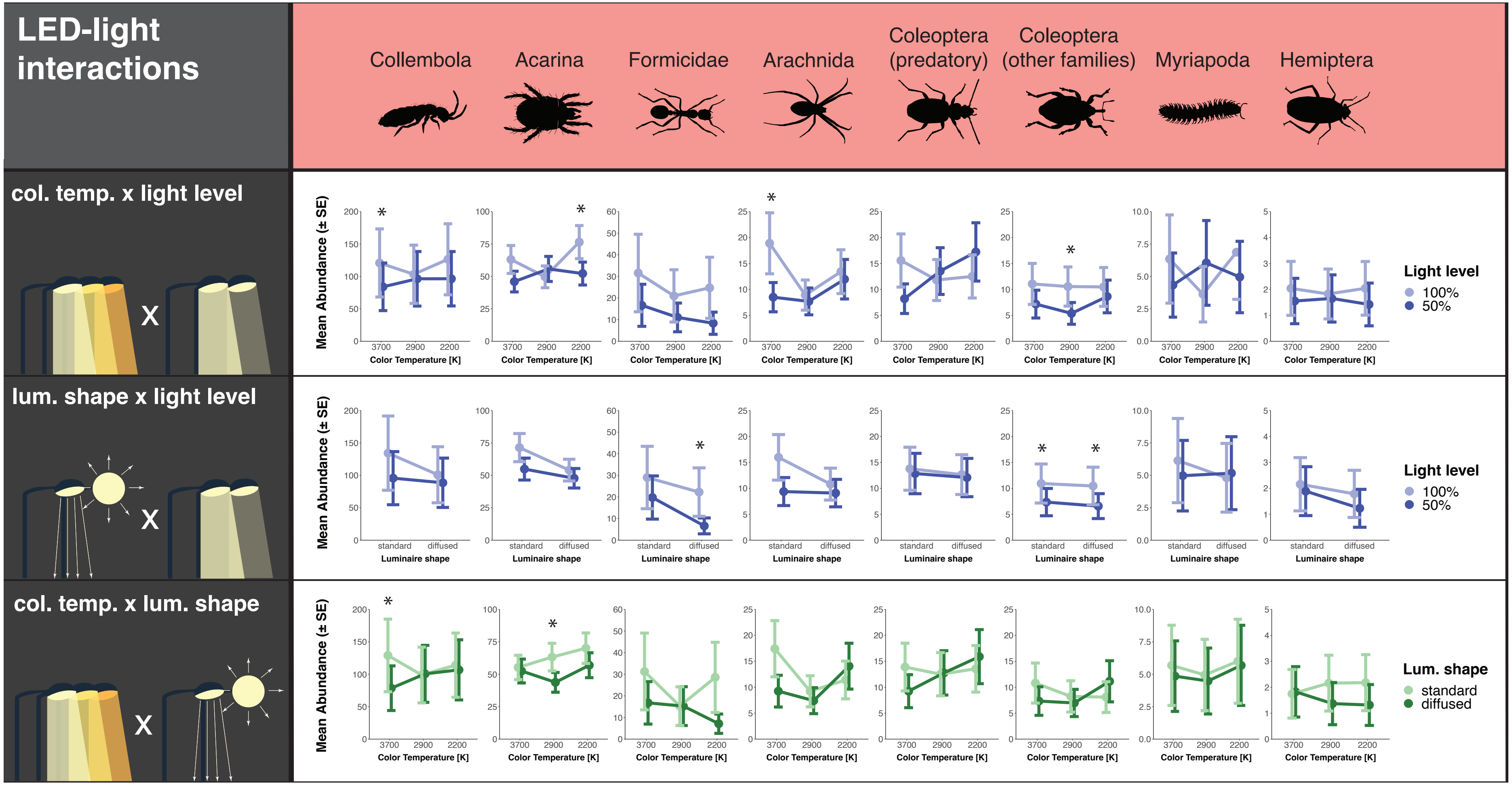

Figure S9. Estimated marginal means for the interactions of three luminaire characteristics (color temperature, light level, luminaire shape) on the abundance of ground-dwelling arthropod taxa. The interactions between the light levels and temperatures averaged across both luminaire shapes (A); the interactions between the light levels and luminaire shapes averaged across the three color temperatures (B) and the interactions between the luminaire shapes and the color temperatures averaged across the two light levels (C). The error bars show the standard error (SE). Significant interactions ($p<0.05$) are indicated by an asterisk (*).

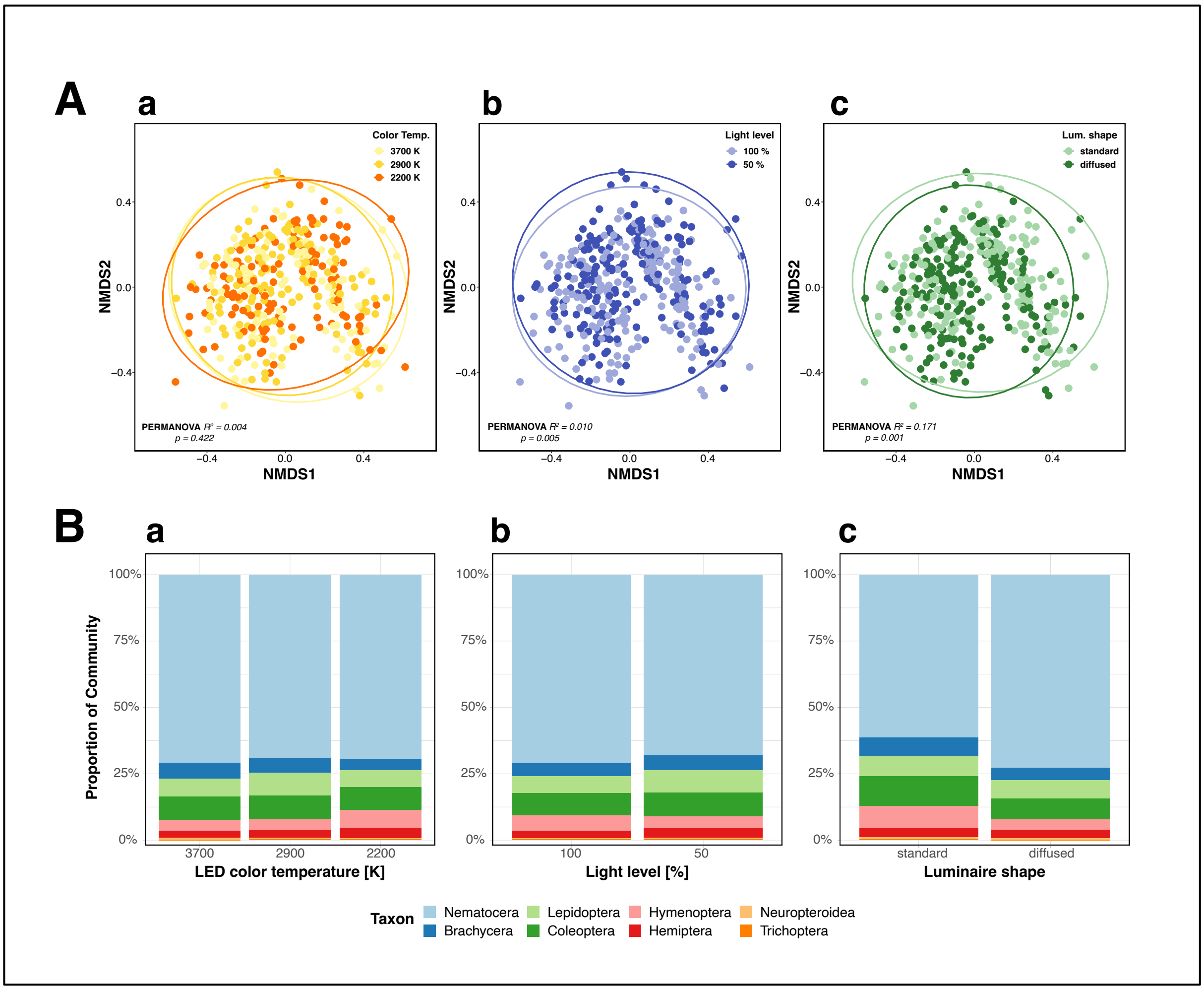

Figure S10. (A) Non-metric multidimensional caling (NMDS) of flight-active insect taxa grouped into orders (Nematocera, Lepidoptera, Brachycera, Coleoptera, Hymenoptera, Hemiptera, Neuropteroidea, Trichoptera) for (a) three LED color temperatures (4000 K, 3000 K, 2200 K), (b) two light intensity levels (100%, 50%), and (c) two luminaire shapes (standard, diffused). The circles show the 95% ellipsoids. PERMANOVA results are displayed in the left bottom corner with the R2 and p value. (B) The mean proportional composition of flight-active insect taxa grouped into orders (Nematocera, Lepidoptera, Brachycera, Coleoptera, Hymenoptera, Hemiptera, Neuropteroidea, Trichoptera) as a function of (a) three LED color temperature (4000 K, 3000 K and 2200 K), (b) two light intensity levels (100%, 50%), and (c) two luminaire shape (standard, diffused).

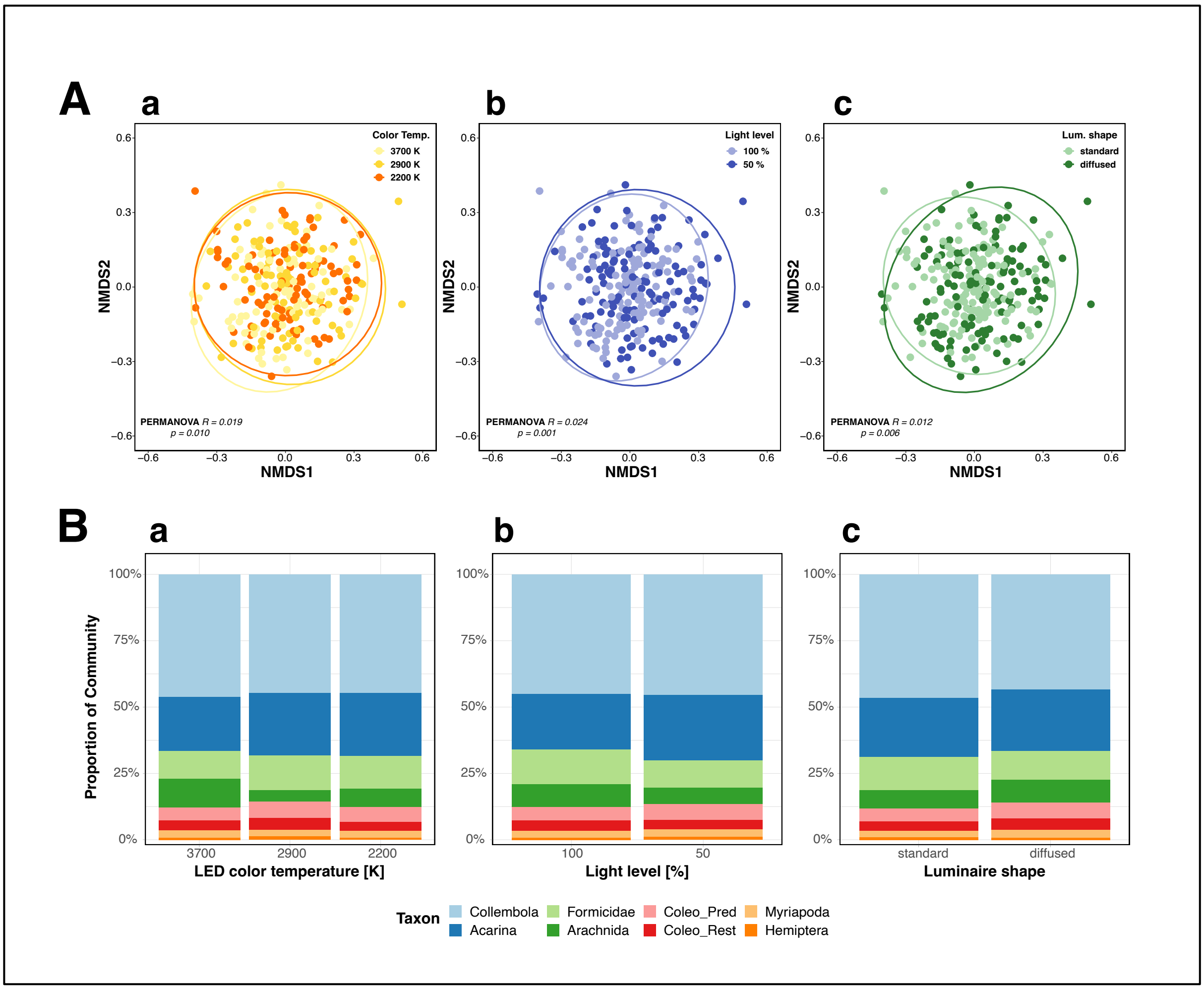

Figure S11. (A) Non-metric multidimensional caling (NMDS) of ground-dwelling arthropod taxa grouped into orders (Collembola, Acarina, Formicidae, Arachnida, Coleoptera (predatory), Coleoptera (rest excluding predatory species), Myriapoda, Hemiptera) for (a) three LED color temperatures (4000 K, 3000 K, 2200 K), (b) two light intensity levels (100%, 50%), and (c) two luminaire shapes (standard, diffused). The circles show the 95% ellipsoids. PERMANOVA results are displayed in the left bottom corner with the R2 and p value. (B) The mean proportional composition of ground-dwelling arthropod taxa grouped into orders (Collembola, Acarina, Formicidae, Arachnida, Coleoptera (predatory), Coleoptera (rest excluding predatory species), Myriapoda, Hemiptera) as a function of (a) three LED color temperature (4000 K, 3000 K and 2200 K), (b) two light intensity levels (100%, 50%), and (c) two luminaire shape (standard, diffused).

Supplementary tables

Table S12. Regression results (ANOVA table) for three groups (flight-active insects & ground-dwelling arthropods, flight-active insects and ground-dwelling arthropods). The explanatory variables are control (2 levels: dark or light), LED color temperature (3 levels), light intensity (2 levels), and luminaire shape (2 levels).
Statistical levels of significance: *** < 0.001 ** < 0.01, * < 0.05

**Flight-active insects & ground-dwelling arthropods**					
Term	*ChiSq*	*Df*	*p-value*	*R2*	*AIC*
(Intercept)	1153.23	1	**< .001*** **		
control	390.19	1	**< .001*** **		
Color temperature	0.54	2	0.77		
Light level	13.75	1	**< .001*** **		
Lum. shape	57.44	1	**< .001*** **	0.54	2000

**Flight-active insects**					
Term	*ChiSq*	*Df*	*p-value*	*R2*	*AIC*
(Intercept)	1529.09	1	**< .001*** **		
control	588.38	1	**< .001*** **		
Color temperature	0.42	2	0.81		
Light level	9.29	1	**0.002** **		
Lum. shape	138.84	1	**< .001*** **	0.74	1118

**Ground-dwelling arthropods**					
Term	*ChiSq*	*Df*	*p-value*	*R2*	*AIC*
(Intercept)	947.62	1	**< .001*** **		
control	23.20	1	**< .001*** **		
Color temperature	2.98	2	0.23		
Light level	8.53	1	**0.003** **		
Lum. shape	3.28	1	0.07	0.58	399

Table S13. Regression results (ANOVA table) for eight flight-active insect taxa. The explanatory variables are control (2 levels: dark or light), LED color temperature (3 levels), light intensity (2 levels), and luminaire shape (2 levels).
Statistical levels of significance: *** < 0.001 ** < 0.01, * < 0.05

**Nematocera**					
Term	*ChiSq*	*Df*	*p-value*	*R2*	*AIC*
(Intercept)	822.52	1	**< .001***		
control	726.11	1	**< .001***		
Color temperature	0.17	2	0.92		
Light level	8.69	1	**0.003**		
Lum. shape	155.21	1	**< .001***	0.87	983

**Lepidoptera**					
Term	*ChiSq*	*Df*	*p-value*	*R2*	*AIC*
(Intercept)	66.38	1	**< .001***		
control	177.33	1	**< .001***		
Color temperature	10.55	2	**0.005**		
Light level	0.80	1	0.37		
Lum. shape	77.29	1	**< .001***	0.79	1058

**Brachycera**					
Term	*ChiSq*	*Df*	*p-value*	*R2*	*AIC*
(Intercept)	140.04	1	**< .001***		
control	227.75	1	**< .001***		
Color temperature	2.82	2	0.24		
Light level	3.55	1	0.06		
Lum. shape	19.70	1	**< .001***	0.77	969

**Coleoptera**					
Term	*ChiSq*	*Df*	*p-value*	*R2*	*AIC*
(Intercept)	44.52	1	**< .001***		
control	178.30	1	**< .001***		
Color temperature	0.37	2	0.83		
Light level	4.59	1	**0.03***		
Lum. shape	65.36	1	**< .001***	0.81	960

**Hymenoptera**					
Term	*ChiSq*	*Df*	*p-value*	*R2*	*AIC*
(Intercept)	20.81	1	**< .001*** **		
control	156.15	1	**< .001*** **		
Color temperature	5.50	2	0.06		
Light level	4.63	1	**0.03***		
Lum. shape	4.54	1	**0.03***	0.81	1059

**Hemiptera**					
Term	*ChiSq*	*Df*	*p-value*	*R2*	*AIC*
(Intercept)	9.04	1	**0.003****		
control	47.34	1	**< .001*** **		
Color temperature	4.56	2	0.10		
Light level	0.76	1	0.38		
Lum. shape	25.50	1	**< .001*** **	0.80	1082

**Neuropteroidea**					
Term	*ChiSq*	*Df*	*p-value*	*R2*	*AIC*
(Intercept)	2.71	1	0.10		
control	24.24	1	**< .001*** **		
Color temperature	6.57	2	0.04		
Light level	0.36	1	0.55		
Lum. shape	31.44	1	**< .001*** **	0.63	930

**Trichoptera**					
Term	*ChiSq*	*Df*	*p-value*	*R2*	*AIC*
(Intercept)	6.29	1	0.01*		
control	12.26	1	**< .001*** **		
Color temperature	1.07	2	0.59		
Light level	1.35	1	0.24		
Lum. shape	9.57	1	**0.002****	0.59	782

Table S14. Regression results (ANOVA table) for eight ground-dwelling arthropod taxa. The explanatory variables are control (2 levels: dark or light), LED color temperature (3 levels), light intensity (2 levels), and luminaire shape (2 levels).
Statistical levels of significance: *** < 0.001 ** < 0.01, * < 0.05

**Collembola**					
Term	*ChiSq*	*Df*	*p-value*	*R2*	*AIC*
(Intercept)	125.16	1	**< .001*** **		
control	10.61	1	**< .001*** **		
Color temperature	0.63	2	0.73		
Light level	4.26	1	**0.04***		
Lum. shape	2.36	1	0.12	0.60	665

**Acarina**					
Term	*ChiSq*	*Df*	*p-value*	*R2*	*AIC*
(Intercept)	825.53	1	**< .001*** **		
control	9.09	1	**0.003****		
Color temperature	2.39	2	0.30		
Light level	3.18	1	0.07		
Lum. shape	3.92	1	**0.048***	0.20	670

**Formicidae**					
Term	*ChiSq*	*Df*	*p-value*	*R2*	*AIC*
(Intercept)	40.46	1	**< .001*** **		
control	9.45	1	**0.002****		
Color temperature	1.23	2	0.54		
Light level	4.58	1	**0.03***		
Lum. shape	3.29	1	0.07	0.80	791

**Arachnida**					
Term	*ChiSq*	*Df*	*p-value*	*R2*	*AIC*
(Intercept)	111.37	1	**< .001*** **		
control	16.00	1	**< .001*** **		
Color temperature	3.93	2	0.14		
Light level	2.98	1	0.08		
Lum. shape	1.08	1	0.30	0.58	746

**Coleoptera (predatory)**					
Term	*ChiSq*	*Df*	*p-value*	*R2*	*AIC*
(Intercept)	90.41	1	**< .001*** **		
control	11.54	1	**< .001*** **		
Color temperature	1.26	2	0.53		
Light level	0.10	1	0.76		
Lum. shape	0.17	1	0.68	0.72	521

**Coleoptera (other families)**					
Term	*ChiSq*	*Df*	*p-value*	*R2*	*AIC*
(Intercept)	74.39	1	**< .001*** **		
control	4.94	1	**0.03***		
Color temperature	1.85	2	0.40		
Light level	9.51	1	**0.002****		
Lum. shape	0.22	1	0.64	0.51	633

**Myriapoda**					
Term	*ChiSq*	*Df*	*p-value*	*R2*	*AIC*
(Intercept)	20.47	1	**< .001*** **		
control	8.40	1	**0.003****		
Color temperature	1.69	2	0.43		
Light level	0.30	1	0.58		
Lum. shape	0.65	1	0.42	0.65	651

**Hemiptera**					
Term	*ChiSq*	*Df*	*p-value*	*R2*	*AIC*
(Intercept)	13.68	1	**< .001*** **		
control	0.51	1	0.47		
Color temperature	0.02	2	0.99		
Light level	1.25	1	0.26		
Lum. shape	2.05	1	0.15	0.40	681

Table S15. Pairwise comparison of control vs. light treatments (Tukey post hoc test). Two levels of controls (light, dark) to explain mean arthropod abundance of three groups (flight-active insects & ground-dwelling arthropods, flight-active insects and ground-dwelling arthropods).

**Flight-active insects & ground-dwelling arthropods**					
**Light vs. Dark (control)**					
Contrasts	*Difference*	*Std. error*	*df*	*t-ratio*	*p-value*
light - dark	2.38	0.09	37.20	25.65	**< .001*** **

**Flight-active insects**					
**Light vs. Dark (control)**					
Contrasts	*Difference*	*Std. error*	*df*	*t-ratio*	*p-value*
light - dark	3.79	0.11	40.76	33.16	**< .001*** **

**Ground-dwelling arthropods**					
**Light vs. Dark (control)**					
Contrasts	*Difference*	*Std. error*	*df*	*t-ratio*	*p-value*
light - dark	0.47	0.13	33.35	3.62	**< .001*** **

Table S16. Pairwise comparison of control vs. light treatments (Tukey post hoc test). Two levels of controls (light, dark) to explain mean arthropod abundance of eight flight-active insect taxa.

**Nematocera**					
**Light vs. Dark (control)**					
Contrasts	*Difference*	*Std. error*	*df*	*t-ratio*	*p-value*
light - dark	4.52	0.12	39.22	36.99	**< .001*** **

**Lepidoptera**					
**Light vs. Dark (control)**					
Contrasts	*Difference*	*Std. error*	*df*	*t-ratio*	*p-value*
light - dark	3.19	0.15	39.17	21.34	**< .001*** **

**Brachycera**					
**Light vs. Dark (control)**					
Contrasts	*Difference*	*Std. error*	*df*	*t-ratio*	*p-value*
light - dark	2.81	0.14	39.20	20.45	**< .001*** **

**Coleoptera**					
**Light vs. Dark (control)**					
Contrasts	*Difference*	*Std. error*	*df*	*t-ratio*	*p-value*
light - dark	2.05	0.11	39.38	18.65	**< .001*****

**Hymenoptera**					
**Light vs. Dark (control)**					
Contrasts	*Difference*	*Std. error*	*df*	*t-ratio*	*p-value*
light - dark	2.35	0.16	39.36	14.63	**< .001*****

**Hemiptera**					
**Light vs. Dark (control)**					
Contrasts	*Difference*	*Std. error*	*df*	*t-ratio*	*p-value*
light - dark	1.45	0.16	39.26	9.12	**< .001*****

**Neuropteroidea**					
**Light vs. Dark (control)**					
Contrasts	*Difference*	*Std. error*	*df*	*t-ratio*	*p-value*
light - dark	0.96	0.10	39.79	9.70	**< .001*****

**Trichoptera**					
**Light vs. Dark (control)**					
Contrasts	*Difference*	*Std. error*	*df*	*t-ratio*	*p-value*
light - dark	0.66	0.14	40.28	4.75	**< .001*****

Table S17. Pairwise comparison of control vs. light treatments (Tukey post hoc test). Two levels of controls (light, dark) to explain mean arthropod abundance of eight ground-dwelling arthropod taxa.

**Collembola**					
**Light vs. Dark (control)**					
Contrasts	*Difference*	*Std. error*	*df*	*t-ratio*	*p-value*
light - dark	0.35	0.15	32.98	2.29	**0.03***

**Acarina**					
**Light vs. Dark (control)**					
Contrasts	*Difference*	*Std. error*	*df*	*t-ratio*	*p-value*
light - dark	0.21	0.14	32.79	1.51	0.14

**Formicidae**					
**Light vs. Dark (control)**					
Contrasts	*Difference*	*Std. error*	*df*	*t-ratio*	*p-value*
light - dark	1.24	0.46	33.81	2.70	**0.011***

**Arachnida**					
**Light vs. Dark (control)**					
Contrasts	*Difference*	*Std. error*	*df*	*t-ratio*	*p-value*
light - dark	0.86	0.25	33.44	3.45	**0.002****

**Coleo_Rest**					
**Light vs. Dark (control)**					
Contrasts	*Difference*	*Std. error*	*df*	*t-ratio*	*p-value*
light - dark	0.15	0.16	33.14	0.92	0.37

**Coleo_Pred**					
**Light vs. Dark (control)**					
Contrasts	*Difference*	*Std. error*	*df*	*t-ratio*	*p-value*
light - dark	0.79	0.23	33.68	3.43	**0.002****

**Myriapoda**					
**Light vs. Dark (control)**					
Contrasts	*Difference*	*Std. error*	*df*	*t-ratio*	*p-value*
light - dark	0.41	0.16	33.09	2.53	**0.02**

**Hemiptera**					
**Light vs. Dark (control)**					
Contrasts	*Difference*	*Std. error*	*df*	*t-ratio*	*p-value*
light - dark	-0.003	0.18	33.15	-0.02	0.99

Table S18. Regression results (ANOVA table) **for** three groups (flight-active insects & ground-dwelling arthropods, flight-active insects and ground-dwelling arthropods). The explanatory variables light color (3 levels), light intensity (2 levels), light shape (2 levels), and all pairwise interactions between light variables (i.e., color*intensity, color*shape, intensity*shape).
Statistical levels of significance: *** < 0.001 ** < 0.01, * < 0.05

**Flight-active insects & ground-dwelling arthropods**					
Term	*ChiSq*	*Df*	*p-value*	*R2*	*AIC*
(Intercept)	3199.31	1	**< .001*** **		
Color temperature	0.55	2	0.76		
Light level	5.79	1	0.02*		
Lum. shape	14.00	1	**< .001*** **		
Temp. : Level	2.15	2	0.34		
Temp. : Shape	0.92	2	0.63		
Level : Shape	0.32	1	0.57	0.14	1590

**Flight-active insects**					
Term	*ChiSq*	*Df*	*p-value*	*R2*	*AIC*
(Intercept)	1745.78	1	**< .001*** **		
Color temperature	0.06	2	0.97		
Light level	5.55	1	0.02*		
Lum. shape	37.87	1	**< .001*** **		
Temp. : Level	1.03	2	0.60		
Temp. : Shape	0.52	2	0.77		
Level : Shape	0.06	1	0.80	0.34	1006

**Ground-dwelling arthropods**					
Term	*ChiSq*	*Df*	*p-value*	*R2*	*AIC*
(Intercept)	115.23	1	**< .001*** **		
Color temperature	1.24	2	0.54		
Light level	0.58	1	0.45		
Lum. shape	0.61	1	0.44		
Temp. : Level	1.20	2	0.55		
Temp. : Shape	4.04	2	0.13		
Level : Shape	0.88	1	0.35	0.57	582

Table S19. Regression results (ANOVA table) **for** eight flight-active insect groups. The explanatory variables light color (3 levels), light intensity (2 levels), light shape (2 levels), and all pairwise interactions between light variables (i.e., color*intensity, color*shape, intensity*shape).
Statistical levels of significance: *** < 0.001 ** < 0.01, * < 0.05

**Nematocera**					
Term	*ChiSq*	*Df*	*p-value*	*R2*	*AIC*
(Intercept)	718.22	1	**< .001*** **		
Color temperature	0.22	2	0.90		
Light level	5.67	1	**0.02***		
Lum. shape	40.46	1	**< .001*** **		
Temp. : Level	1.89	2	0.39		
Temp. : Shape	0.80	2	0.67		
Level : Shape	0.05	1	0.82	0.64	849

**Lepidoptera**					
Term	*ChiSq*	*Df*	*p-value*	*R2*	*AIC*
(Intercept)	91.56	1	**< .001*** **		
Color temperature	1.71	2	0.42		
Light level	2.00	1	0.16		
Lum. shape	16.40	1	**< .001*** **		
Temp. : Level	1.51	2	0.47		
Temp. : Shape	0.31	2	0.86		
Level : Shape	0.17	1	0.68	0.64	941

**Brachycera**					
Term	*ChiSq*	*Df*	*p-value*	*R2*	*AIC*
(Intercept)	141.19	1	**< .001*** **		
Color temperature	7.86	2	**0.02***		
Light level	3.77	1	0.05		
Lum. shape	5.96	1	**0.01***		
Temp. : Level	8.92	2	**0.01***		
Temp. : Shape	0.40	2	0.82		
Level : Shape	0.20	1	0.66	0.57	863

**Coleoptera**					
Term	*ChiSq*	*Df*	*p-value*	*R2*	*AIC*
(Intercept)	64.61	1	**< .001*** **		
Color temperature	3.36	2	0.19		
Light level	1.56	1	0.21		
Lum. shape	24.99	1	**< .001*** **		
Temp. : Level	1.04	2	0.60		
Temp. : Shape	2.37	2	0.31		
Level : Shape	2.03	1	0.15	0.79	804

**Hymenoptera**					
Term	*ChiSq*	*Df*	*p-value*	*R2*	*AIC*
(Intercept)	21.20	1	**< .001*****		
Color temperature	10.14	2	**0.006****		
Light level	5.45	1	**0.02***		
Lum. shape	4.19	1	0.04		
Temp. : Level	1.66	2	0.44		
Temp. : Shape	1.20	2	0.55		
Level : Shape	0.27	1	0.60	0.78	923

**Hemiptera**					
Term	*ChiSq*	*Df*	*p-value*	*R2*	*AIC*
(Intercept)	26.24	1	**< .001*****		
Color temperature	3.28	2	0.19		
Light level	0.12	1	0.73		
Lum. shape	10.87	1	**< .001*****		
Temp. : Level	1.05	2	0.59		
Temp. : Shape	1.14	2	0.57		
Level : Shape	0.49	1	0.49	0.38	1278

**Neuropteroidea**					
Term	*ChiSq*	*Df*	*p-value*	*R2*	*AIC*
(Intercept)	6.69	1	**0.009****		
Color temperature	0.78	2	0.68		
Light level	0.03	1	0.86		
Lum. shape	5.83	1	**0.02***		
Temp. : Level	2.84	2	0.24		
Temp. : Shape	1.23	2	0.54		
Level : Shape	1.77	1	0.18	0.67	805

**Trichoptera**					
Term	*ChiSq*	*Df*	*p-value*	*R2*	*AIC*
(Intercept)	9.71	1	0.002**		
Color temperature	3.10	2	0.21		
Light level	1.40	1	0.24		
Lum. shape	0.41	1	0.52		
Temp. : Level	4.25	2	0.12		
Temp. : Shape	1.06	2	0.59		
Level : Shape	0.31	1	0.57	0.60	718

Table S20. Regression results (ANOVA table) **for** eight ground-dwelling arthropod groups. The explanatory variables light color (3 levels), light intensity (2 levels), light shape (2 levels), and all pairwise interactions between light variables (i.e., color*intensity, color*shape, intensity*shape). Statistical levels of significance: *** < 0.001 ** < 0.01, * < 0.05

**Collembola**					
Term	*ChiSq*	*Df*	*p-value*	*R2*	*AIC*
(Intercept)	115.23	1	**< .001*** **		
Color temperature	1.24	2	0.54		
Light level	0.58	1	0.45		
Lum. shape	0.61	1	0.44		
Temp. : Level	1.20	2	0.55		
Temp. : Shape	4.04	2	0.13		
Level : Shape	0.88	1	0.35	0.57	582

**Acarina**					
Term	*ChiSq*	*Df*	*p-value*	*R2*	*AIC*
(Intercept)	481.05	1	**< .001*** **		
Color temperature	6.40	2	**0.04***		
Light level	2.45	1	0.12		
Lum. shape	1.97	1	0.16		
Temp. : Level	4.74	2	0.09		
Temp. : Shape	1.58	2	0.45		
Level : Shape	0.48	1	0.49	0.22	584

**Formicidae**					
Term	*ChiSq*	*Df*	*p-value*	*R2*	*AIC*
(Intercept)	17.56	1	**< .001*** **		
Color temperature	0.61	2	0.74		
Light level	3.41	1	0.06		
Lum. shape	1.49	1	0.22		
Temp. : Level	0.26	2	0.88		
Temp. : Shape	1.97	2	0.37		
Level : Shape	0.99	1	0.32	0.80	689

**Arachnida**					
Term	*ChiSq*	*Df*	*p-value*	*R2*	*AIC*
(Intercept)	58.02	1	**< .001*** **		
Color temperature	2.26	2	0.32		
Light level	0.02	1	0.88		
Lum. shape	0.01	1	0.94		
Temp. : Level	2.22	2	0.33		
Temp. : Shape	2.66	2	0.26		
Level : Shape	0.72	1	0.39	0.56	662

**Coleoptera (predatory)**					
Term	*ChiSq*	*Df*	*p-value*	*R2*	*AIC*
(Intercept)	55.96	1	**< .001*** **		
Color temperature	0.10	2	0.95		
Light level	0.75	1	0.39		
Lum. shape	0.15	1	0.70		
Temp. : Level	4.50	2	0.11		
Temp. : Shape	1.51	2	0.47		
Level : Shape	0.00	1	0.95	0.70	462

**Coleoptera (other families)**					
Term	*ChiSq*	*Df*	*p-value*	*R2*	*AIC*
(Intercept)	55.79	1	**< .001*** **		
Color temperature	1.22	2	0.54		
Light level	0.72	1	0.40		
Lum. shape	1.75	1	0.19		
Temp. : Level	2.02	2	0.36		
Temp. : Shape	4.85	2	0.09		
Level : Shape	0.05	1	0.82	0.54	559

**Myriapoda**					
Term	*ChiSq*	*Df*	*p-value*	*R2*	*AIC*
(Intercept)	17.27	1	**< .001*** **		
Color temperature	5.52	2	0.06		
Light level	0.48	1	0.49		
Lum. shape	0.51	1	0.48		
Temp. : Level	8.33	2	**0.02***		
Temp. : Shape	0.09	2	0.95		
Level : Shape	1.02	1	0.31	0.67	561

**Hemiptera**					
Term	*ChiSq*	*Df*	*p-value*	*R2*	*AIC*
(Intercept)	7.17	1	0.007**		
Color temperature	0.61	2	0.74		
Light level	1.09	1	0.30		
Lum. shape	0.78	1	0.38		
Temp. : Level	0.23	2	0.89		
Temp. : Shape	1.17	2	0.56		
Level : Shape	0.22	1	0.64	0.42	604

Table S21. Pairwise comparison (Tukey post hoc test) of light treatments (LED temperature, light level, luminaire shape) to explain mean arthropod abundance of three groups (flight-active insects & ground-dwelling arthropods, flight-active insects and ground-dwelling arthropods). The explanatory variables are light color (3 levels), light intensity (2 levels), light shape (2 levels), and all pairwise interactions between light variables (i.e., color*intensity, color*shape, intensity*shape).
Statistical levels of significance: *** < 0.001 ** < 0.01, * < 0. 05.

**Flight-active insects & ground-dwelling arthropods**					
**Light Temperatures**					
Contrasts	*Difference*	*Std. error*	*df*	*t-ratio*	*p-value*
2200 K - 2900 K	-0.05	0.08	23.62	-0.65	> 0.99
2200 K - 3700 K	-0.02	0.08	23.12	-0.25	> 0.99
2900 K - 3700 K	0.03	0.08	24.44	0.40	> 0.99
**Light Levels**					
100% - 50%	-0.25	0.06	23.74	-4.01	**< .001*** **
**Luminaire shape**					
Standard - Diffusor	0.53	0.06	23.73	8.43	**< .001*** **

**Flight-active insects**					
**Light Temperatures**					
Contrasts	*Difference*	*Std. error*	*df*	*t-ratio*	*p-value*
2200 K - 2900 K	0.03	0.10	24.26	0.34	> 0.99
2200 K - 3700 K	-0.03	0.10	23.00	-0.29	> 0.99
2900 K - 3700 K	-0.03	0.10	24.20	-0.63	> 0.99
**Light Levels**					
100% - 50%	-0.25	0.08	23.76	-3.03	**0.006** **
**Luminaire shape**					
Standard - Diffusor	0.98	0.08	23.68	11.80	**< .001*** **

**Ground-dwelling arthropods**					
**Light Temperatures**					
Contrasts	*Difference*	*Std. error*	*df*	*t-ratio*	*p-value*
2200 K - 2900 K	-0.19	0.12	23.57	-1.66	0.33
2200 K - 3700 K	-0.04	0.12	23.76	-0.37	0.71
2900 K - 3700 K	0.15	0.12	24.28	1.28	0.43
**Light Levels**					
100% - 50%	-0.28	0.10	23.87	-2.98	**0.007** **
**Luminaire shape**					
Standard - Diffusor	-0.18	0.10	23.93	-1.84	0.08

Table S22. Pairwise comparison (Tukey post hoc test) of light treatments (LED temperature, light level, luminaire shape) to explain mean arthropod abundance of eight flight-active insect taxa. The explanatory variables are light color (3 levels), light intensity (2 levels), light shape (2 levels), and all pairwise interactions between light variables (i.e., color*intensity, color*shape, intensity*shape). Statistical levels of significance: *** < 0.001 ** < 0.01, * < 0. 05.

**Nematocera**					
**Light Temperatures**					
Contrasts	*Difference*	*Std. error*	*df*	*t-ratio*	*p-value*
2200 K - 2900 K	-0.04	0.10	24.38	-0.35	> 0.99
2200 K - 3700 K	-0.04	0.10	23.42	-0.41	> 0.99
2900 K - 3700 K	-0.01	0.10	24.37	-0.05	> 0.99
**Light Levels**					
100% - 50%	-0.26	0.08	24.04	-3.14	**0.004****
**Luminaire shape**					
Standard - Diffusor	1.14	0.08	23.99	13.57	**< .001*****

**Lepidoptera**					
**Light Temperatures**					
Contrasts	*Difference*	*Std. error*	*df*	*t-ratio*	*p-value*
2200 K - 2900 K	0.43	0.15	24.50	2.82	**0.03***
2200 K - 3700 K	0.29	0.15	23.74	1.93	0.13
2900 K - 3700 K	-0.14	0.15	24.49	-0.90	0.38
**Light Levels**					
100% - 50%	-0.08	0.12	24.23	-0.68	0.502
**Luminaire shape**					
Standard - Diffusor	0.98	0.12	24.22	7.87	**< .001*****

**Brachycera**					
**Light Temperatures**					
Contrasts	*Difference*	*Std. error*	*df*	*t-ratio*	*p-value*
2200 K - 2900 K	0.19	0.12	24.44	1.66	0.33
2200 K - 3700 K	0.03	0.12	23.56	0.27	0.79
2900 K - 3700 K	-0.16	0.12	24.41	-1.39	0.35
**Light Levels**					
100% - 50%	-0.20	0.09	23.9	-2.10	**0.046***
**Luminaire shape**					
Standard - Diffusor	0.45	0.09	24.08	4.81	**< .001*****

**Coleoptera**					
**Light Temperatures**					
Contrasts	*Difference*	*Std. error*	*df*	*t-ratio*	*p-value*
2200 K - 2900 K	-0.01	0.08	24.33	-0.17	> 0.99
2200 K - 3700 K	-0.05	0.08	23.18	-0.63	> 0.99
2900 K - 3700 K	-0.04	0.08	24.27	-0.46	> 0.99
**Light Levels**					
100% - 50%	-0.17	0.07	23.87	-2.54	**0.02***
**Luminaire shape**					
Standard - Diffusor	0.66	0.07	23.82	9.80	**< .001*****

**Hymenoptera**					
**Light Temperatures**					
Contrasts	*Difference*	*Std. error*	*df*	*t-ratio*	*p-value*
2200 K - 2900 K	-0.18	0.12	24.41	-1.55	0.26
2200 K - 3700 K	-0.36	0.12	23.46	-3.13	**0.01***
2900 K - 3700 K	-0.18	0.12	24.37	-1.57	0.26
**Light Levels**					
100% - 50%	-0.24	0.09	24.04	-2.60	**0.02***
**Luminaire shape**					
Standard - Diffusor	0.24	0.09	24.01	2.58	**0.02***

**Hemiptera**					
**Light Temperatures**					
Contrasts	*Difference*	*Std. error*	*df*	*t-ratio*	*p-value*
2200 K - 2900 K	-0.21	0.15	24.26	-1.40	0.35
2200 K - 3700 K	-0.31	0.15	23.00	-2.05	0.16
2900 K - 3700 K	-0.09	0.15	24.20	-0.63	0.54
**Light Levels**					
100% - 50%	-0.10	0.12	23.75	-0.85	0.4
**Luminaire shape**					
Standard - Diffusor	0.59	0.12	23.68	4.84	**< .001*****

**Neuropteroidea**					
**Light Temperatures**					
Contrasts	*Difference*	*Std. error*	*df*	*t-ratio*	*p-value*
2200 K - 2900 K	0.22	0.09	24.34	2.47	0.06
2200 K - 3700 K	0.16	0.09	23.26	1.86	0.15
2900 K - 3700 K	-0.05	0.09	24.30	-0.62	0.54
**Light Levels**					
100% - 50%	0.05	0.07	23.93	0.66	0.52
**Luminaire shape**					
Standard - Diffusor	0.41	0.07	23.87	5.75	**< .001*** **

**Trichoptera**					
**Light Temperatures**					
Contrasts	*Difference*	*Std. error*	*df*	*t-ratio*	*p-value*
2200 K - 2900 K	-0.05	0.13	24.55	-0.38	0.99
2200 K - 3700 K	-0.14	0.13	23.84	-1.07	0.88
2900 K - 3700 K	-0.09	0.13	24.54	-0.69	0.99
**Light Levels**					
100% - 50%	-0.12	0.10	24.30	-1.16	0.259
**Luminaire shape**					
Standard - Diffusor	0.33	0.10	24.29	3.16	**0.004** **

Table S23. Pairwise comparison (Tukey post hoc test) of light treatments (LED temperature, light level, luminaire shape) to explain mean arthropod abundance of eight ground-dwelling arthropod taxa. The explanatory variables are light color (3 levels), light intensity (2 levels), light shape (2 levels), and all pairwise interactions between light variables (i.e., color*intensity, color*shape, intensity*shape). Statistical levels of significance: *** < 0.001 ** < 0.01, * < 0. 05.

**Collembola**					
**Light Temperatures**					
Contrasts	*Difference*	*Std. error*	*df*	*t-ratio*	*p-value*
2200 K - 2900 K	-0.10	0.13	23.15	-0.75	> 0.99
2200 K - 3700 K	-0.09	0.13	23.46	-0.68	> 0.99
2900 K - 3700 K	0.01	0.14	24.21	0.07	> 0.99
**Light Levels**					
100% - 50%	-0.23	0.11	23.61	-2.11	**0.046***
**Luminaire shape**					
Standard - Diffusor	-0.18	0.11	23.75	-1.63	0.12

**Acarina**					
**Light Temperatures**					
Contrasts	*Difference*	*Std. error*	*df*	*t-ratio*	*p-value*
2200 K - 2900 K	-0.18	0.12	22.91	-1.48	0.45
2200 K - 3700 K	-0.16	0.12	23.24	-1.29	0.45
2900 K - 3700 K	0.02	0.12	24.09	0.19	0.85
**Light Levels**					
100% - 50%	-0.19	0.10	23.43	-1.91	0.069
**Luminaire shape**					
Standard - Diffusor	-0.20	0.10	23.6	-2.05	0.051

**Formicidae**					
**Light Temperatures**					
Contrasts	*Difference*	*Std. error*	*df*	*t-ratio*	*p-value*
2200 K - 2900 K	0.05	0.46	23.92	0.10	> 0.99
2200 K - 3700 K	0.44	0.46	23.96	0.94	> 0.99
2900 K - 3700 K	0.39	0.46	24.10	0.84	> 0.99
**Light Levels**					
100% - 50%	-0.74	0.38	23.99	-1.97	0.06
**Luminaire shape**					
Standard - Diffusor	-0.63	0.38	24.00	-1.67	0.11

**Arachnida**					
**Light Temperatures**					
Contrasts	*Difference*	*Std. error*	*df*	*t-ratio*	*p-value*
2200 K - 2900 K	-0.39	0.24	23.67	-1.62	0.35
2200 K - 3700 K	0.01	0.24	23.82	0.02	0.98
2900 K - 3700 K	0.39	0.24	24.26	1.63	0.35
**Light Levels**					
100% - 50%	-0.33	0.20	23.92	-1.66	0.11
**Luminaire shape**					
Standard - Diffusor	-0.20	0.20	23.96	-1.00	0.33

**Coleoptera (other families)**					
**Light Temperatures**					
Contrasts	*Difference*	*Std. error*	*df*	*t-ratio*	*p-value*
2200 K - 2900 K	-0.20	0.15	23.36	-1.37	0.55
2200 K - 3700 K	-0.06	0.15	23.63	-0.39	0.7
2900 K - 3700 K	0.15	0.15	24.27	0.97	0.68
**Light Levels**					
100% - 50%	-0.39	0.12	23.76	-3.18	**0.004****
**Luminaire shape**					
Standard - Diffusor	-0.07	0.12	23.86	-0.57	0.58

**Coleoptera (predatory)**					
**Light Temperatures**					
Contrasts	*Difference*	*Std. error*	*df*	*t-ratio*	*p-value*
2200 K - 2900 K	-0.14	0.22	23.84	-0.63	> 0.99
2200 K - 3700 K	-0.24	0.22	23.92	-1.08	0.87
2900 K - 3700 K	-0.10	0.22	24.17	-0.45	> 0.99
**Light Levels**					
100% - 50%	-0.05	0.18	23.98	-0.30	0.77
**Luminaire shape**					
Standard - Diffusor	-0.07	0.18	23.99	-0.38	0.71

**Myriapoda**					
**Light Temperatures**					
Contrasts	*Difference*	*Std. error*	*df*	*t-ratio*	*p-value*
2200 K - 2900 K	-0.18	0.14	23.33	-1.27	0.65
2200 K - 3700 K	-0.09	0.14	23.60	-0.62	> 0.99
2900 K - 3700 K	0.09	0.14	24.26	0.63	> 0.99
**Light Levels**					
100% - 50%	-0.06	0.12	23.73	-0.51	0.62
**Luminaire shape**					
Standard - Diffusor	-0.09	0.12	23.85	-0.74	0.47

**Hemiptera**					
**Light Temperatures**					
Contrasts	*Difference*	*Std. error*	*df*	*t-ratio*	*p-value*
2200 K - 2900 K	0.01	0.17	23.45	0.05	> 0.99
2200 K - 3700 K	0.03	0.18	23.68	0.15	> 0.99
2900 K - 3700 K	0.02	0.18	24.30	0.10	> 0.99
**Light Levels**					
100% - 50%	-0.16	0.14	23.82	-1.08	0.29
**Luminaire shape**					
Standard - Diffusor	-0.19	0.14	23.87	-1.34	0.19

Table S24. Pairwise comparison (Tukey post-hoc test) of the interactions between 3 levels of LED colors (3700K, 2900K, 2200K), 2 light levels (100%, 50%), 2 luminaire shapes (standard and diffusor) to explain mean arthropod abundance of three groups (flight-active insects & ground-dwelling arthropods, flight-active insects and ground-dwelling arthropods). The explanatory variables are light color (3 levels), light intensity (2 levels), light shape (2 levels), and all pairwise interactions between light variables (i.e., color*intensity, color*shape, intensity*shape).
Statistical levels of significance: *** < 0.001 ** < 0.01, * < 0. 05.

**Flight-active insects & ground-dwelling arthropods**					
**Light Temp : Light Level**					
Contrasts	*Difference*	*Std. error*	*df*	*t-ratio*	*p-value*
2200 K: 100% - 50%	-0.33	0.11	22.32	-3.11	**0.005****
2900 K: 100% - 50%	-0.12	0.11	25.06	-1.11	0.28
3700 K: 100% - 50%	-0.30	0.11	23.93	-2.74	**0.01***
**Light shape : Light Level**					
Diffused: 100% - 50%	-0.22	0.09	24.14	-2.42	**0.02***
Standard: 100% - 50%	-0.29	0.09	23.31	-3.25	**0.004****
**Light Temp : Light shape**					
2200 K: Standard - Diffusor	0.50	0.11	22.35	4.65	**< .001*****
2900 K: Standard - Diffusor	0.61	0.11	25.04	5.58	**< .001*****
3700 K: Standard - Diffusor	0.48	0.11	23.93	4.36	**< .001*****

**Flight-active insects**					
**Light Temp : Light Level**					
Contrasts	*Difference*	*Std. error*	*df*	*t-ratio*	*p-value*
2200 K: 100% - 50%	-0.36	0.14	22.88	-2.52	**0.02***
2900 K: 100% - 50%	-0.22	0.15	25.45	-1.47	0.15
3700 K: 100% - 50%	-0.18	0.14	23.04	-1.26	0.22
**Light shape : Light Level**					
Diffused: 100% - 50%	-0.26	0.12	23.36	-2.22	**0.04***
Standard: 100% - 50%	-0.25	0.12	24.18	-2.07	**0.049***
**Light Temp : Light shape**					
2200 K: Standard - Diffusor	0.93	0.14	22.92	6.48	**< .001*****
2900 K: Standard - Diffusor	1.06	0.15	25.29	7.23	**< .001*****
3700 K: Standard - Diffusor	0.96	0.14	22.92	6.72	**< .001*****

Ground-dwelling arthropods					
**Light Temp : Light Level**					
Contrasts	*Difference*	*Std. error*	*df*	*t-ratio*	*p-value*
2200 K: 100% - 50%	-0.28	0.16	23.01	-1.69	0.1
2900 K: 100% - 50%	-0.10	0.17	24.13	-0.60	0.55
3700 K: 100% - 50%	-0.48	0.17	24.44	-2.86	**0.009****
**Light shape : Light Level**					
Diffused: 100% - 50%	-0.20	0.14	24.51	-1.46	0.16
Standard: 100% - 50%	-0.37	0.13	23.23	-2.76	**0.01***
**Light Temp : Light shape**					
2200 K: Standard - Diffusor	-0.13	0.16	23.03	-0.80	0.43
2900 K: Standard - Diffusor	-0.10	0.17	24.13	-0.63	0.53
3700 K: Standard - Diffusor	-0.29	0.17	24.55	-1.76	0.09

Table S25. Pairwise comparison (Tukey post-hoc test) of the interactions between 3 levels of LED colors (3700K, 2900K, 2200K), 2 light levels (100%, 50%), 2 luminaire shapes (standard and diffusor) to explain mean arthropod abundance of eight flight-active insect groups. The explanatory variables are light color (3 levels), light intensity (2 levels), light shape (2 levels), and all pairwise interactions between light variables (i.e., color*intensity, color*shape, intensity*shape).
Statistical levels of significance: *** < 0.001 ** < 0.01, * < 0. 05.

Nematocera					
**Light Temp : Light Level**					
Contrasts	*Difference*	*Std. error*	*df*	*t-ratio*	*p-value*
2200 K: 100% - 50%	-0.38	0.15	23.41	-2.61	**0.02****
2900 K: 100% - 50%	-0.11	0.15	25.37	-0.72	0.48
3700 K: 100% - 50%	-0.31	0.15	23.45	-2.13	**0.04****
**Light shape : Light Level**					
Diffused: 100% - 50%	-0.28	0.12	23.85	-2.40	**0.03****
Standard: 100% - 50%	-0.25	0.12	24.21	-2.05	**0.05****
**Light Temp : Light shape**					
2200 K: Standard - Diffusor	1.04	0.15	23.40	7.20	**< .001*****
2900 K: Standard - Diffusor	1.23	0.15	25.32	8.37	**< .001*****
3700 K: Standard - Diffusor	1.15	0.15	23.39	7.93	**< .001*****

**Lepidoptera**					
**Light Temp : Light Level**					
Contrasts	*Difference*	*Std. error*	*df*	*t-ratio*	*p-value*
2200 K: 100% - 50%	-0.30	0.22	23.75	-1.40	0.18
2900 K: 100% - 50%	0.01	0.22	25.28	0.06	0.95
3700 K: 100% - 50%	0.03	0.22	23.76	0.15	0.88
**Light shape : Light Level**					
Diffused: 100% - 50%	-0.14	0.18	24.24	-0.77	0.45
Standard: 100% - 50%	-0.03	0.18	24.21	-0.19	0.85
**Light Temp : Light shape**					
2200 K: Standard - Diffusor	0.96	0.22	23.74	4.43	**< .001*** **
2900 K: Standard - Diffusor	1.07	0.22	25.26	4.98	**< .001*** **
3700 K: Standard - Diffusor	0.91	0.22	23.74	4.22	**< .001*** **

**Brachycera**					
**Light Temp : Light Level**					
Contrasts	*Difference*	*Std. error*	*df*	*t-ratio*	*p-value*
2200 K: 100% - 50%	-0.32	0.16	23.55	-1.98	0.06
2900 K: 100% - 50%	-0.45	0.16	25.31	-2.76	**0.01** **
3700 K: 100% - 50%	0.20	0.16	23.58	1.21	0.24
**Light shape : Light Level**					
Diffused: 100% - 50%	-0.24	0.13	24.01	-1.77	0.09
Standard: 100% - 50%	-0.15	0.13	24.20	-1.13	0.27
**Light Temp : Light shape**					
2200 K: Standard - Diffusor	0.42	0.16	23.54	2.56	**0.02***
2900 K: Standard - Diffusor	0.54	0.16	25.27	3.28	**0.003** **
3700 K: Standard - Diffusor	0.41	0.16	23.54	2.49	**0.02***

**Coleoptera**					
**Light Temp : Light Level**					
Contrasts	*Difference*	*Std. error*	*df*	*t-ratio*	*p-value*
2200 K: 100% - 50%	-0.26	0.12	23.14	-2.27	**0.03***
2900 K: 100% - 50%	-0.15	0.12	25.41	-1.26	0.22
3700 K: 100% - 50%	-0.10	0.12	23.23	-0.86	0.39
**Light shape : Light Level**					
Diffused: 100% - 50%	-0.08	0.10	23.58	-0.79	0.44
Standard: 100% - 50%	-0.27	0.10	24.18	-2.78	**0.01***
**Light Temp : Light shape**					
2200 K: Standard - Diffusor	0.77	0.12	23.14	6.59	**< .001*** **
2900 K: Standard - Diffusor	0.70	0.12	25.32	5.90	**< .001*** **
3700 K: Standard - Diffusor	0.52	0.12	23.13	4.48	**< .001*** **

**Hymenoptera**					
**Light Temp : Light Level**					
Contrasts	*Difference*	*Std. error*	*df*	*t-ratio*	*p-value*
2200 K: 100% - 50%	-0.39	0.16	23.45	-2.39	**0.03***
2900 K: 100% - 50%	-0.25	0.16	25.33	-1.54	0.14
3700 K: 100% - 50%	-0.09	0.16	23.49	-0.57	0.58
**Light shape : Light Level**					
Diffused: 100% - 50%	-0.29	0.13	23.89	-2.21	**0.04***
Standard: 100% - 50%	-0.20	0.13	24.20	-1.46	0.16
**Light Temp : Light shape**					
2200 K: Standard - Diffusor	0.34	0.16	23.44	2.06	0.05
2900 K: Standard - Diffusor	0.30	0.16	25.28	1.80	0.09
3700 K: Standard - Diffusor	0.10	0.16	23.43	0.61	0.55

**Hemiptera**					
**Light Temp : Light Level**					
Contrasts	*Difference*	*Std. error*	*df*	*t-ratio*	*p-value*
2200 K: 100% - 50%	0.001	0.21	22.88	0.003	0.998
2900 K: 100% - 50%	-0.28	0.22	25.44	-1.31	0.2
3700 K: 100% - 50%	-0.03	0.21	23.03	-0.15	0.88
**Light shape : Light Level**					
Diffused: 100% - 50%	-0.19	0.17	23.36	-1.10	0.28
Standard: 100% - 50%	-0.02	0.17	24.18	-0.11	0.91
**Light Temp : Light shape**					
2200 K: Standard - Diffusor	0.71	0.21	22.91	3.39	**0.003****
2900 K: Standard - Diffusor	0.65	0.22	25.29	3.02	**0.006****
3700 K: Standard - Diffusor	0.41	0.21	22.91	1.96	0.06

**Neuropteroidea**					
**Light Temp : Light Level**					
Contrasts	*Difference*	*Std. error*	*df*	*t-ratio*	*p-value*
2200 K: 100% - 50%	-0.12	0.12	23.23	-0.98	0.34
2900 K: 100% - 50%	0.16	0.13	25.40	1.25	0.22
3700 K: 100% - 50%	0.11	0.12	23.30	0.86	0.4
**Light shape : Light Level**					
Diffused: 100% - 50%	0.14	0.10	23.66	1.41	0.17
Standard: 100% - 50%	-0.05	0.10	24.19	-0.47	0.64
**Light Temp : Light shape**					
2200 K: Standard - Diffusor	0.44	0.12	23.22	3.55	**0.002****
2900 K: Standard - Diffusor	0.49	0.13	25.32	3.93	**< .001*****
3700 K: Standard - Diffusor	0.30	0.12	23.21	2.46	**0.022***

**Trichoptera**					
**Light Temp : Light Level**					
Contrasts	*Difference*	*Std. error*	*df*	*t-ratio*	*p-value*
2200 K: 100% - 50%	-0.30	0.18	23.85	-1.69	0.11
2900 K: 100% - 50%	-0.24	0.18	25.28	-1.32	0.20
3700 K: 100% - 50%	0.18	0.18	23.86	1.00	0.33
**Light shape : Light Level**					
Diffused: 100% - 50%	-0.06	0.15	24.37	-0.42	0.68
Standard: 100% - 50%	-0.18	0.15	24.22	-1.21	0.24
**Light Temp : Light shape**					
2200 K: Standard - Diffusor	0.19	0.18	23.84	1.07	0.3
2900 K: Standard - Diffusor	0.45	0.18	25.26	2.52	**0.02***
3700 K: Standard - Diffusor	0.34	0.18	23.84	1.88	0.07

Table S26. Pairwise comparison (Tukey post-hoc test) of the interactions between 3 levels of LED colors (3700K, 2900K, 2200K), 2 light levels (100%, 50%), 2 luminaire shapes (standard and diffusor) to explain mean arthropod abundance of eight ground-dwelling arthropod groups. The explanatory variables are light color (3 levels), light intensity (2 levels), light shape (2 levels), and all pairwise interactions between light variables (i.e., color*intensity, color*shape, intensity*shape).
Statistical levels of significance: *** < 0.001 ** < 0.01, * < 0. 05.

**Collembola**					
**Light Temp : Light Level**					
Contrasts	*Difference*	*Std. error*	*df*	*t-ratio*	*p-value*
2200 K: 100% - 50%	-0.27	0.19	22.35	-1.43	0.17
2900 K: 100% - 50%	-0.07	0.19	23.98	-0.36	0.72
3700 K: 100% - 50%	-0.36	0.19	24.42	-1.86	0.08
**Light shape : Light Level**					
Diffused: 100% - 50%	-0.13	0.16	24.51	-0.82	0.42
Standard: 100% - 50%	-0.34	0.15	22.70	-2.19	**0.04***
**Light Temp : Light shape**					
2200 K: Standard - Diffusor	-0.07	0.19	22.39	-0.35	0.73
2900 K: Standard - Diffusor	0.02	0.19	23.98	0.09	0.93
3700 K: Standard - Diffusor	-0.49	0.19	24.78	-2.54	**0.02***

**Acarina**					
**Light Temp : Light Level**					
Contrasts	*Difference*	*Std. error*	*df*	*t-ratio*	*p-value*
2200 K: 100% - 50%	-0.38	0.17	22.03	-2.23	**0.04***
2900 K: 100% - 50%	0.11	0.17	23.86	0.65	0.52
3700 K: 100% - 50%	-0.31	0.18	24.30	-1.76	0.09
**Light shape : Light Level**					
Diffused: 100% - 50%	-0.12	0.14	24.41	-0.84	0.41
Standard: 100% - 50%	-0.26	0.14	22.43	-1.87	0.07
**Light Temp : Light shape**					
2200 K: Standard - Diffusor	-0.20	0.17	22.06	-1.21	0.24
2900 K: Standard - Diffusor	-0.36	0.17	23.86	-2.07	**0.049***
3700 K: Standard - Diffusor	-0.05	0.18	24.77	-0.28	0.78

**Formicidae**					
**Light Temp : Light Level**					
Contrasts	*Difference*	*Std. error*	*df*	*t-ratio*	*p-value*
2200 K: 100% - 50%	-1.01	0.65	23.76	-1.56	0.13
2900 K: 100% - 50%	-0.60	0.65	24.07	-0.92	0.37
3700 K: 100% - 50%	-0.61	0.65	24.14	-0.94	0.36
**Light shape : Light Level**					
Diffused: 100% - 50%	-1.12	0.53	24.17	-2.09	**0.047***
Standard: 100% - 50%	-0.37	0.53	23.81	-0.69	0.49
**Light Temp : Light shape**					
2200 K: Standard - Diffusor	-1.29	0.65	23.76	-1.98	0.06
2900 K: Standard - Diffusor	0.001	0.65	24.07	0.002	1.00
3700 K: Standard - Diffusor	-0.60	0.65	24.15	-0.91	0.37

**Arachnida**					
**Light Temp : Light Level**					
Contrasts	*Difference*	*Std. error*	*df*	*t-ratio*	*p-value*
2200 K: 100% - 50%	-0.11	0.34	23.20	-0.32	0.76
2900 K: 100% - 50%	-0.13	0.34	24.14	-0.38	0.7
3700 K: 100% - 50%	-0.74	0.34	24.39	-2.16	**0.04***
**Light shape : Light Level**					
Diffused: 100% - 50%	-0.16	0.28	24.45	-0.57	0.58
Standard: 100% - 50%	-0.49	0.28	23.38	-1.79	0.09
**Light Temp : Light shape**					
2200 K: Standard - Diffusor	0.19	0.34	23.22	0.58	0.57
2900 K: Standard - Diffusor	-0.19	0.34	24.14	-0.57	0.57
3700 K: Standard - Diffusor	-0.59	0.34	24.45	-1.72	0.1

**Coleoptera (other families)**					
**Light Temp : Light Level**					
Contrasts	*Difference*	*Std. error*	*df*	*t-ratio*	*p-value*
2200 K: 100% - 50%	-0.17	0.21	22.67	-0.84	0.41
2900 K: 100% - 50%	-0.59	0.21	24.07	-2.81	**0.01***
3700 K: 100% - 50%	-0.39	0.21	24.47	-1.83	0.08
**Light shape : Light Level**					
Diffused: 100% - 50%	-0.41	0.17	24.55	-2.39	**0.03***
Standard: 100% - 50%	-0.36	0.17	22.96	-2.11	**0.046***
**Light Temp : Light shape**					
2200 K: Standard - Diffusor	0.29	0.21	22.70	1.39	0.18
2900 K: Standard - Diffusor	-0.15	0.21	24.07	-0.70	0.49
3700 K: Standard - Diffusor	-0.35	0.21	24.70	-1.64	0.11

**Coleoptera (predatory)**					
**Light Temp : Light Level**					
Contrasts	*Difference*	*Std. error*	*df*	*t-ratio*	*p-value*
2200 K: 100% - 50%	0.30	0.31	23.57	0.96	0.35
2900 K: 100% - 50%	0.13	0.31	24.11	0.40	0.69
3700 K: 100% - 50%	-0.59	0.31	24.24	-1.87	0.07
**Light shape : Light Level**					
Diffused: 100% - 50%	-0.04	0.26	24.29	-0.17	0.87
Standard: 100% - 50%	-0.06	0.25	23.66	-0.25	0.80
**Light Temp : Light shape**					
2200 K: Standard - Diffusor	0.15	0.31	23.58	0.48	0.64
2900 K: Standard - Diffusor	0.02	0.31	24.11	0.06	0.95
3700 K: Standard - Diffusor	-0.37	0.31	24.26	-1.19	0.25

**Myriapoda**					
**Light Temp : Light Level**					
Contrasts	*Difference*	*Std. error*	*df*	*t-ratio*	*p-value*
2200 K: 100% - 50%	-0.28	0.20	22.61	-1.39	0.18
2900 K: 100% - 50%	0.42	0.20	24.06	2.06	0.05
3700 K: 100% - 50%	-0.32	0.21	24.47	-1.56	0.13
**Light shape : Light Level**					
Diffused: 100% - 50%	0.06	0.17	24.54	0.35	0.73
Standard: 100% - 50%	-0.18	0.16	22.91	-1.09	0.29
**Light Temp : Light shape**					
2200 K: Standard - Diffusor	-0.05	0.20	22.65	-0.23	0.82
2900 K: Standard - Diffusor	-0.08	0.20	24.06	-0.40	0.69
3700 K: Standard - Diffusor	-0.13	0.21	24.71	-0.65	0.52

Hemiptera					
**Light Temp : Light Level**					
Contrasts	*Difference*	*Std. error*	*df*	*t-ratio*	*p-value*
2200 K: 100% - 50%	-0.23	0.24	22.83	-0.93	0.36
2900 K: 100% - 50%	-0.06	0.25	24.11	-0.25	0.8
3700 K: 100% - 50%	-0.17	0.25	24.49	-0.70	0.49
**Light shape : Light Level**					
Diffused: 100% - 50%	-0.22	0.20	24.55	-1.09	0.29
Standard: 100% - 50%	-0.09	0.20	23.08	-0.44	0.67
**Light Temp : Light shape**					
2200 K: Standard - Diffusor	-0.32	0.24	22.83	-1.29	0.21
2900 K: Standard - Diffusor	-0.29	0.25	24.11	-1.15	0.26
3700 K: Standard - Diffusor	0.03	0.25	24.62	0.11	0.91

Table S27. Results of permutational multivariate analysis of variance (PERMANOVA) assessing the effects of three LED lighting properties (color temperature, light level and luminaire shape) on flight-active insect community composition. The analysis was conducted using the Bray-Curtis dissimilarity matrix. Statistical levels of significance: *** < 0.001 ** < 0.01, * < 0. 05.

**Flight-active insects**					
Variable	*Df*	*Sum of Sqs*	*R2*	*F-value*	*p-value*
Color temp.	2	0.24	0.004	0.99	0.42
Light level	1	0.60	0.01	4.87	**0.002****
Lum. Shape	1	9.86	0.17	79.67	**0.001*****
Residuals	380	47.03	0.81		
Total	384	57.73	1.00		

Table S28. Results of permutational multivariate analysis of variance (PERMANOVA) assessing the effects of three LED lighting properties (color temperature, light level and luminaire shape) on ground-dwelling arthropod community composition. The analysis was conducted using the Bray-Curtis dissimilarity matrix. Statistical levels of significance: *** < 0.001 ** < 0.01, * < 0. 05.

**Ground-dwelling arthropods**					
Variable	*Df*	*Sum of Sqs*	*R2*	*F-value*	*p-value*
Color temp.	2	0.51	0.02	2.57	**0.01***
Light level	1	0.66	0.02	6.61	**0.001*****
Lum. Shape	1	0.34	0.01	3.38	**0.006****
Residuals	257	25.50	0.94		
Total	261	27.00	1.00		

Table S29. Light technical information for all light treatments (LED color, dimming levels and luminaire shapes). Correction factors were applied to standardize the luminous flux for all light treatments (luminaires with and without diffusors, all dimmlevels, and light colors). All luminaires were set to a luminous flux of 1500 lumens.

**Luminaire model**
Izylum1, 20 LED with lens optics

**Light color temperature (Kelvin)**		
**Color description**	**Manufacturer specification**	**Lab measurement**
Amber	2200 K	2155 K
Warm white	3000 K	2889 K
Neutral white	4000 K	3699 K

**Luminous flux (Lumen)**		
**Color description**	**Lab measurement**	**Experiment (with corrections)**
Amber	2231 lm	1500 lm
Warm white	1935 lm	1500 lm
Neutral white	1938 lm	1500 lm

Table S30. Results of the Moran's I test for spatial autocorrelation of residuals from the linear mixed-effects model analyzing arthropod abundance captured at streetlights with different combinations of LED-light properties (log-transformed). The analysis was conducted using a spatial weights matrix ('W' style) based on neighbors within a 40-meter threshold distance.

Variable	*Moran's I*	*Z-score*	*Expectation*	*p-value*
Residuals from LMM	-0.016	-1.019	-0.003	0.846
